# Low Density Receptor-Related Protein 1 Interactions With the Extracellular Matrix: More Than Meets the Eye

**DOI:** 10.3389/fcell.2019.00031

**Published:** 2019-03-15

**Authors:** Ewa E. Bres, Andreas Faissner

**Affiliations:** Department of Cell Morphology and Molecular Neurobiology, Ruhr University Bochum, Bochum, Germany

**Keywords:** low density receptor-related protein 1, tissue plasminogen activator, integrins, extracellular matrix, migration, matrix remodeling

## Abstract

The extracellular matrix (ECM) is a biological substrate composed of collagens, proteoglycans and glycoproteins that ensures proper cell migration and adhesion and keeps the cell architecture intact. The regulation of the ECM composition is a vital process strictly controlled by, among others, proteases, growth factors and adhesion receptors. As it appears, ECM remodeling is also essential for proper neuronal and glial development and the establishment of adequate synaptic signaling. Hence, disturbances in ECM functioning are often present in neurodegenerative diseases like Alzheimer’s disease. Moreover, mutations in ECM molecules are found in some forms of epilepsy and malfunctioning of ECM-related genes and pathways can be seen in, for example, cancer or ischemic injury. Low density lipoprotein receptor-related protein 1 (Lrp1) is a member of the low density lipoprotein receptor family. Lrp1 is involved not only in ligand uptake, receptor mediated endocytosis and lipoprotein transport—functions shared by low density lipoprotein receptor family members—but also regulates cell surface protease activity, controls cellular entry and binding of toxins and viruses, protects against atherosclerosis and acts on many cell signaling pathways. Given the plethora of functions, it is not surprising that Lrp1 also impacts the ECM and is involved in its remodeling. This review focuses on the role of Lrp1 and some of its major ligands on ECM function. Specifically, interactions with two Lrp1 ligands, integrins and tissue plasminogen activator are described in more detail.

## Introduction

### Lrp1: A Hidden Multitasker

**FIGURE 1:**
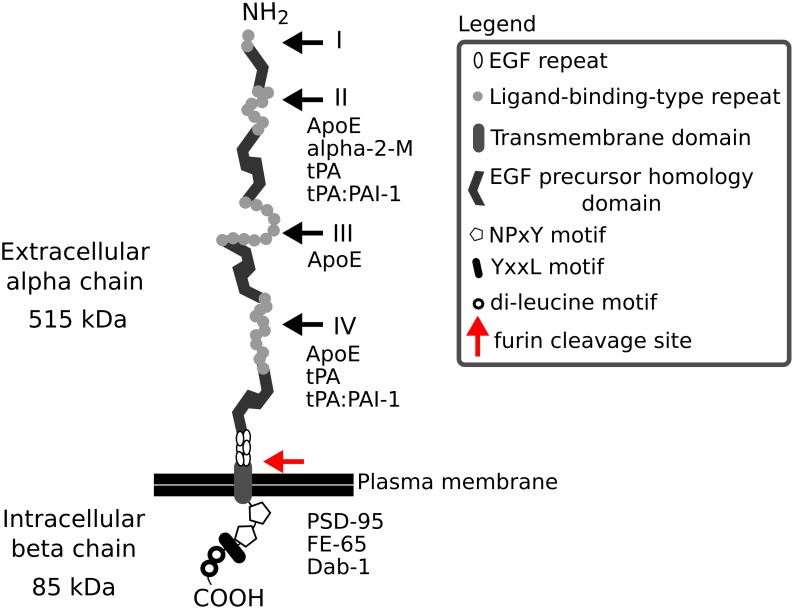
The structure of Lrp1. Lrp1 is cleaved by furin (red arrow) in the Golgi complex and afterwards is transported to the cell membrane. The resulting mature Lrp1 receptor consists of a 515 kDa alpha chain and an 85 kDa beta chain bound to each other non-covalently. The alpha chain consists of ligand-binding-type repeats forming four clusters (I–IV) that are rich in cysteine. The clusters contain 2, 8, 10, and 11 repeats, respectively. The clusters II and IV are responsible for the majority of ligand binding to Lrp1. The ligand-binding clusters are separated by 1–4 EGF homology domains, with cysteine-rich EGF repeats. The intracellular beta chain consists of two NPxY motifs, one YxxL motif and two di-leucine motifs that have been associated with the endocytotic functions of Lrp1. The beta chain also interacts with scaffolding proteins like PSD-95, Dab-1, and FE-65. Both the extra- and intracellular chain can act independently of each other, when the alpha chain is shed as a soluble Lrp1 and the beta chain translocates to the nucleus and activates gene transcription and signaling cascades. α-2-M, α-2-macroglobulin; ApoE, apolipoprotein E; COOH, carboxy terminal; Dab-1, disabled 1; EGF, epidermal growth factor; NH_2_, amino terminal; PAI-1, plasminogen activator inhibitor 1; PSD-95, postsynaptic density protein 95; tPA, tissue plasminogen activator.

Low density lipoprotein receptor-related protein-1 (Lrp1), also known as CD91 or α-2-macroglobulin (α-2-M) receptor, is a member of the low density lipoprotein receptor family and is expressed in various tissues including liver, adipose tissue, lungs and brain. The receptor, with a mass of 600 kDa, during its biosynthesis, undergoes a furin-mediated proteolytical cleavage in the Golgi apparatus. This cleavage results in two, non-covalently bound polypeptide subunits—an 85 kDa membrane-bound C-terminal fragment (the light β chain) and a 515 kDa N-terminal fragment located extracellularly (the heavy α chain)—that form the mature Lrp1 (Figure 1). The Lrp1 α chain contains 4 ligand binding complement-like repeat clusters separated by epidermal growth factor (EGF) repeats. Clusters II and IV are considered to be responsible for the majority of ligand binding (Herz, 2001; Herz and Strickland, 2001; Croy et al., 2003; Meijer et al., 2007) and have previously been shown to display minor differences regarding the kinetics of the interactions but to be also highly similar in their ligand binding properties (duplication of the domains) (Neels et al., 1999). As suggested by Huang et al. (1999), each ligand-binding domain of Lrp1 presents very different charge densities and hydrophobic patches. These differences in turn lead to varying receptor-ligand interactions and are responsible for a distinct ligand specificity of each cluster, despite similar backbone folds. Because Lrp1 interacts with a wide variety of protein ligands (Table 1), it is necessary to study each domain individually to elucidate its exact ligand-binding capacities. Interestingly, receptor-associated protein (RAP) binds to the ligand-binding clusters and completely blocks interactions with all known Lrp1 ligands (Bu et al., 1994).

**Table 1:** Representative molecules interacting with the Lrp1 receptor.

Molecule and function		Reference
α1-antitrypsin (A1AT or A1PI)	Member of the serpin superfamily, inhibits various proteases, regulates enzymes produced by inflammatory cells like neutrophil elastase	Poller et al., 1995
α1-antitrypsin:trypsin complexes	Serpin-enzyme complex	Kounnas et al., 1996
α-2 macroglobulin (α-2-M)	Member of the α-2 globulin family. Protease inhibitor, inhibits a wide range of proteinases	Ashcom et al., 1990; Strickland et al., 1990
Amidoglycosides: gentamicin, polymixcin B	Antibiotics used to treat various bacterial infections	Moestrup et al., 1995
Amyloid β peptide	Peptide derived from amyloid precursor protein processing. Main component of amyloid plaques found in Alzheimer’s patients	Kang et al., 2000; Shibata et al., 2000
Amyloid precursor protein (APP)	Integral membrane protein, during its proteolysis the amyloid β peptide is generated	Kounnas et al., 1995a; Ulery et al., 2000
Annexin VI	Member of the calcium-dependent membrane and phospholipid binding proteins; co-receptor of Lrp1, involved in endocytosis processes, interacts with α-2-M	Ling et al., 2004
Apolipoprotein E (ApoE)/ApoE-containing lipoproteins	Fat-binding protein produced by astrocytes, essential for the catabolism of lipoproteins and their transport; main cholesterol carrier in the brain	Herz et al., 1988; Beisiegel et al., 1989; Hayashi et al., 2007
Aprotinin	Single-chain globular polypeptide derived from bovine lungs; inhibits serine proteases	Hussain et al., 1999
Bone morphogenic factor 4 (BMP4) BMP-binding endothelial cell precursor-derived regulator	Growth and differentiation factor; important for neurogenesis, bone and cartilage metabolism	Pi et al., 2012
C1s/C1q	Form the complement component C1 complex that initiates the classical pathway of component activation	Storm et al., 1997
C4b-binding protein (C4BP)	Inhibitor in the complement system	Spijkers et al., 2008
Calreticulin	Calcium-binding chaperone protein, regulates many cellular processes	Gardai et al., 2005
Cathepsin D	Lysosomal aspartic protease, member of the peptidase A1 family, involved in protein degradation	Derocq et al., 2012
CCN1, cysteine-rich angiogenic inducer 61 (CYR61)	Secreted, matrix-associated signaling protein involved in apoptosis, adhesion, migration and vascular integrity	Juric et al., 2012
Chylomicron remnants	Lipoprotein particles comprising triglycerides, phospholipids, cholesterol, and proteins involved in lipid transport	Rohlmann et al., 1998; Kowal et al., 1989
Coagulation factor VIII	Blood-clotting protein, participates in blood coagulation	Lenting et al., 1999; Saenko et al., 1999
Coagulation factor Xa: tissue factor pathway inhibitor (TFPI) complexes	Coagulation factor X is a serine protease that in its active form (Xa) converts prothrombin into thrombin and plays a role in blood coagulation; TFPI reversibly inhibits factor Xa	Ho et al., 1996
Coagulation factor XIa:nexin complexes	Coagulation factor XI is a serine protease that in its active form (XIa) initiates the intrinsic pathway of blood coagulation by activating factor IX; complexes with nexin-1 inhibit its function	Knauer et al., 2000
Complement component 3	Plays a role in the activation of the classical and alternative complement activation pathways	Meilinger et al., 1999
Connective tissue growth factor (CTGF; CCN2)	Matricellular protein of the extracellular matrix-associated heparin-binding protein family, involved in cell adhesion, migration, and angiogenesis	Segarini et al., 2001; Gao and Brigstock, 2003
Decorin (Dcn)	Member of the small leucine-rich proteoglycan family that impacts the activities of growth factors, regulates extracellular matrix assembly and cell adhesion	Brandan et al., 2006
Disabled 1 (Dab1)	Adaptor protein known to activate Src	Trommsdorff et al., 1998; Gotthardt et al., 2000
FE-65	Adaptor protein involved in APP processing	Trommsdorff et al., 1998; Gotthardt et al., 2000
Fibronectin	Glycoprotein of the extracellular matrix vital for cell differentiation, migration and adhesion	Salicioni et al., 2002
Frizzled-1	G-coupled receptor protein involved in the Wnt pathway	Zilberberg et al., 2004
Glypican-3:Hedgehog complexes	Glypican-3 is a heparan sulfate proteoglycan that impacts embryonic growth by inhibiting the hedgehog signaling pathway	Capurro et al., 2012
Heat shock protein 90, 96, 70	Intracellular chaperon proteins assisting in protein folding	Basu et al., 2001; Tsen et al., 2013
Heparan sulfate proteoglycans (HSPGs)	Glycoproteins containing one or more covalently attached heparan sulfate chains; present at the cell surface and in the extracellular matrix; endocytic and adhesion receptors, regulate cell migration	Wilsie and Orlando, 2003
Hepatic lipase	Lipase involved in lipoprotein metabolism and transport	Kounnas et al., 1995b
HIV-Tat protein	Transactivator of viral genes in cells infected with HIV	Liu et al., 2000
Insulin	Peptide hormone produced by the pancreas that regulates the metabolism of carbohydrates, fats and proteins	Bilodeau et al., 2010
Insulin-like growth factor-binding protein 3 (IGFBP-3)	Protein produced and secreted by the liver, carrier of insulin-like growth factors	Huang et al., 2003
Lactoferrin	Multifunctional protein of the transferrin family with an antibacterial function	Willnow et al., 1992; Meilinger et al., 1995
Leptin	Hormone produced by adipose cells involved in energy balance and neuronal functioning	Liu et al., 2011
Lipoprotein lipase (LPL)	Lipase involved in lipoprotein metabolism and transport	Beisiegel et al., 1991; Chappell et al., 1992
Malaria circumsporozoite protein (CSP)	Secreted protein of the sporozoite stage of the malaria parasite	Shakibaei and Frevert, 1996
Matrix metalloproteinase 2 (MMP-2)	Proteinase involved in the degradation of the extracellular matrix, metastasis	Yang et al., 2001
Matrix metalloproteinase 9 (MMP-9)	Proteinase involved in the degradation of the extracellular matrix, angiogenesis, metastasis	Hahn-Dantona et al., 2001
Matrix metalloproteinase 13 (collagenase-3) (MMP-13)	Proteinase involved in the degradation of the extracellular matrix, angiogenesis, metastasis	Barmina et al., 1999
Metallothionein II	Cysteine-rich low molecular weight metallothionein family member involved in protection against oxidative stress and chemotactic signal transduction	Landowski et al., 2016
Midkine (MDK)	Heparin-binding growth factor induced during mid-gestation involved in cell migration, survival and angiogenesis	Muramatsu et al., 2000; Lee et al., 2012
Minor-group human rhinovirus (HRV2)	Minor group rhinovirus	Hofer et al., 1994
Myelin-associated glycoprotein (MAG)	Cell membrane glycoprotein involved in myelination	Stiles et al., 2013
Myelin basic protein (MBP)	Major protein forming the myelin sheath of oligodendrocytes and Schwann cells	Gaultier et al., 2009
Nexin-1	Member of the serine protease inhibitor (Serpin) superfamily	Crisp et al., 2000; Vaillant et al., 2007
Plasminogen activator inhibitor (PAI-1)	Serpin, Regulator of tPA/uPA activity	Stefansson et al., 1998
Platelet-derived growth factor (PDGF)-BB PDGF receptor (PDGFR) β	PDGF-BB is a dimeric glycoprotein composed of two B subunits and a major growth factor that binds with high affinity to the cell surface receptor PDGFR β	Boucher et al., 2002; Loukinova et al., 2002; Takayama et al., 2005; Zhou et al., 2009
Postsynaptic density protein 95 (PSD-95)	Adaptor protein crucial for synapse stability and coupling to NMDA receptors	Gotthardt et al., 2000; May et al., 2004
Prion protein (PrP)	Cell-surface glycoprotein that upon conversion can cause prion diseases	Sunyach et al., 2003; Taylor and Hooper, 2007
Pregnancy zone protein (PZP):protease complexes	PZP is a member of the α-2 globulin family; protease inhibitor and extracellular chaperone; role in immune regulation during pregnancy	Moestrup et al., 1987; Sanchez et al., 2001
Pro-urokinase	Serine protease, urokinase-type plasminogen activator single-chain zymogen with little intrinsic enzymatic activity	Kounnas et al., 1993
Pseudomonas exotoxin A	Toxin from *Pseudomonas aeruginosa*	Kounnas et al., 1992b
Receptor-associated protein (RAP)	Endoplasmic reticulum resident chaperone glycoprotein, inhibits binding of ligands to low density lipoprotein receptor family members	Herz et al., 1991; Kounnas et al., 1992a
Ricin A	Ribosome-inactivating protein found in the seeds of *Ricinus communis*; potent toxin	Cavallaro et al., 1995
Saporin	Ribosome-inactivating protein found in the seeds of *Saponaria officinalis*; potent toxin	Cavallaro et al., 1993a,b, 1995
Saposin (SAP) precursor	Glycoprotein precursor of saposins (sphingolipid activator proteins) involved in glycosphingolipid catabolism	Hiesberger et al., 1998
Shc	Adaptor protein that becomes phosphorylated on tyrosine residues in response to extracellular stimuli	Barnes et al., 2001
Trigliceride-rich lipoproteins (TLRs)	Main carriers of triglycerides in the blood; involved in lipoprotein metabolism and transport	Mahley and Huang, 2007; Foley et al., 2013
Tissue inhibitors of matrix metalloproteases (TIMPs)	Protease inhibitors of matrix metalloproteinases	Scilabra et al., 2013; Thevenard et al., 2014
Tissue factor pathway inhibitor (TFPI)	Single-chain polypeptide that reversibly inhibits coagulation factor Xa, thereby regulating blood clotting	Warshawsky et al., 1994
TpeL	*Clostridium perfringens* toxin	Schorch et al., 2014
Transforming growth factor-β 1 (TGF-β 1)	Multifunctional growth factor, involved in interactions with extracellular proteins, cell growth, differentiation and vascular remodeling	Huang et al., 2003
Transforming growth factor-β 2 (TGF-β 2)	Multifunctional growth factor, involved in interactions with extracellular proteins, cell growth, differentiation and vascular remodeling	Muratoglu et al., 2011
Thrombospondin 1	Extracellular matrix glycoprotein, member of the thrombospondin family, vital for cell-cell and cell-matrix interactions	Godyna et al., 1995; Mikhailenko et al., 1995
Thrombospondin 2	Extracellular matrix glycoprotein, member of the thrombospondin family, vital for cell-cell and cell-matrix interactions	Meng et al., 2010
Tissue-type plasminogen activator (tPA)	Serine protease mediating the conversion of plasminogen to plasmin and cell signaling	Bu et al., 1992; Zhuo et al., 2000
tPA:PAI-1 complexes	Serine protease–protease inhibitor complex	Orth et al., 1992
tPA:neuroserpin complexes	Serine protease–protease inhibitor complex	Makarova et al., 2003
Thrombin:protein inhibitor C complexes	Serine protease–protease inhibitor complex	Kasza et al., 1997
Thrombin:nexin-1 complexes	Serine protease–protease inhibitor complex	Knauer et al., 1997
Thrombin:antithrombin III complexes	Serine protease–protease inhibitor complex	Kounnas et al., 1996
Thrombin:heparin cofactor II complexes	Serine protease–protease inhibitor complex	Kounnas et al., 1996
Thrombin:PAI-1 complexes	Serine protease–protease inhibitor complex	Stefansson et al., 1996
Trichosanthin	Ribosome-inactivating protein derived from *Trichosanthes kirilowii*	Chan et al., 2000
Urokinase-type plasminogen activator (uPA)	Serine protease, involved in tissue remodeling, wound healing, cell migration	Kounnas et al., 1993
uPA:PAI-1 complexes	Serine protease–protease inhibitor complex	Herz et al., 1992; Nykjaer et al., 1992
uPA:PAI-2 complexes	Serine protease–protease inhibitor complex	Croucher et al., 2006
uPA:C inhibitor complexes	Serine protease–protease inhibitor complex	Kasza et al., 1997
uPA:Nexin-1 complexes	Serine protease–protease inhibitor complex	Conese et al., 1994
Von Willebrand factor (vWF)	Adhesive, glycoprotein involved in blood coagulation and wound healing	Rastegarlari et al., 2012


As shown by Li et al. (2000, 2001), the Lrp1 β chain C-terminus contains motifs, proposed later by Deane et al. (2008) to be involved in generating the rapid endocytotic rate of Lrp1: two NPxY motifs, one YxxL motif and two di-leucine motifs. The C-terminus of Lrp1 interacts with many intracellular ligands (Betts et al., 2008; Guttman et al., 2009) and binds to endocytic and scaffold adaptors like disabled-1, FE-65 and postsynaptic density protein 95 (PSD-95) that link the Lrp1 receptor to membrane-bound proteins such as amyloid precursor protein (APP) (Herz and Chen, 2006; Waldron et al., 2008) and are involved in many cell signaling pathways (Trommsdorff et al., 1998; Gotthardt et al., 2000; May et al., 2004; Pietrzik et al., 2004; Herz et al., 2009; Klug et al., 2011). Lrp1 can additionally undergo an intramembrane proteolysis that results in a shed extracellular Lrp1 fragment and a γ-secretase cleaved intracellular Lrp1 domain. Upon cleavage, the intracellular Lrp1 domain translocates to the cell nucleus and modulates gene expression (May et al., 2002; Zurhove et al., 2008).

Lrp1 is nowadays considered to be a multifunctional receptor: it is involved not only in ligand uptake, receptor-mediated endocytosis, cellular signaling and lipoprotein transport (Herz and Bock, 2002) but also regulates cell surface protease activity (Makarova et al., 2003), controls cellular entry and binding of toxins and viruses (Kounnas et al., 1992b; Hofer et al., 1994; Liu et al., 2000), participates in dendritic cell efferocytosis (Subramanian et al., 2014), protects against atherosclerosis (Boucher and Herz, 2011), is critical for angiogenesis and the maintenance of the blood–brain barrier (BBB) (Polavarapu et al., 2007; Pi et al., 2012; Strickland et al., 2014) and acts on many signaling cascades including the Wnt and Notch pathways (Zilberberg et al., 2004; Lillis et al., 2008; Meng et al., 2010).

In the central nervous system (CNS), Lrp1 is highly expressed not only in neurons, astrocytes and microglia (Bu et al., 1994; Ishiguro et al., 1995; Rebeck et al., 1995; Marzolo et al., 2000; Makarova et al., 2003; Andersen and Willnow, 2006; Kanekiyo et al., 2011; Auderset et al., 2016) but also in brain endothelial cells, vascular smooth muscle cells, pericytes and the choroid plexus (Herz and Bock, 2002).

In the mouse, the complete knock-out of Lrp1 is lethal for the embryos (Herz et al., 1992, 1993), making it challenging to study the role and function of Lrp1 in embryonic as well as adult brain *in vivo*. However, various mutant mouse models generated in the last decades shed more light onto the possible function of Lrp1 in the CNS.

With their studies, May et al. (2004); Liu et al. (2010) and Nakajima et al. (2013), highlight and support earlier findings showing that Lrp1 regulates postsynaptic signaling via interactions with PSD-95 and *N*-methyl-D-aspartate receptor (NMDAR) and is crucial for synaptic transmission (Bacskai et al., 2000; Qiu et al., 2002). The studies of Liu et al. (2010) and Liu et al. (2011) additionally provide evidence for the importance of Lrp1 in maintaining proper brain lipid metabolism and leptin signaling and define Lrp1 as a major apolipoprotein E (ApoE) transport receptor, strengthening the role of Lrp1 in Alzheimer’s disease pathogenesis.

Knock-in mutations in the NPxY2 region lead to a reduced Lrp1 internalization rate (Roebroek et al., 2006; Reekmans et al., 2010; Gordts et al., 2012) and interfere with NMDAR recycling and NMDAR-mediated activation of the ERK1/2 pathway (Martin et al., 2008; Reekmans et al., 2010). Alterations in NMDAR subunits on the cell surface can lead to alterations and deficits in memory processes. Animals harboring the knock-in mutation in the NPxY2 motif display hyperactivity, impaired learning as well as deficits in spatial and reversal learning, similarly to animals with impaired NMDAR signaling (Bannerman et al., 1995; Sakimura et al., 1995).

A recent study from our laboratory discovered that Lrp1 is a novel carrier protein for Lewis X glycans expressed by mouse radial glial cells [neural stem precursor cells (NSPCs)] in the developing and adult CNS (Hennen et al., 2013). With this and a follow-up study we showed that Lrp1 plays a role in the differentiation of NSPCs (Hennen et al., 2013; Safina et al., 2016). The impaired differentiation of Lrp1-lacking NSPCs toward oligodendrocytes is supported by the work of Lin et al. (2017) where deficits in myelination and oligodendrocyte precursor differentiation were observed upon Lrp1 deletion specifically from the oligodendrocyte lineage. According to this study, these impairments are a combined result of altered AKT, sterol regulatory element-binding protein 2 and peroxisome proliferation-associated receptor γ pathways in oligodendrocytes.

### The Extracellular Matrix

The extracellular matrix (ECM) is a biological substrate that is composed of collagens, proteoglycans and glycoproteins. The highly organized honeycomb-like structures of the ECM were first described by Camillo Golgi over a century ago and, since then, the ECM has been found to be essential not only for cell migration, adhesion and structural support but also for proper neuronal and glial development, BBB maturation and function, synaptogenesis and synaptic signaling in the CNS (Faissner et al., 2010; Menezes et al., 2014; Song and Dityatev, 2018). The composition of the ECM is heterogeneous and tissue-specific. In the CNS, the ECM is formed by proteoglycans like heparan sulfate proteoglycans (HSPGs), chondroitin sulfate proteoglycans and glycoproteins including tenascins, laminins, and thrombospondins. During embryonic and early postnatal development, the ECM provides an environment supporting cell migration, differentiation and synapse formation. Here, the ECM forms a loose structure consisting of neuronal neurocan and matures simultaneously with synapses (Pyka et al., 2011). In adult mice, neurocan expression becomes decreased while brevican, aggrecan, and tenascin R become upregulated.

Both ECM components released by neurons and astrocytes are essential for ECM generation (Geissler et al., 2013). Such molecules tend to accumulate especially in ECM structures termed perineuronal nets (PNNs). These consist of chondroitin sulfate proteoglycans, tenascin-R, hyaluronic acid and link proteins. PNNs are found predominantly on the soma and dendrites of parvalbumin-expressing GABAergic neurons (Hartig et al., 1992), however, excitatory pyramidal neurons have also been shown to bear PNNs (Carstens et al., 2016; Lensjø et al., 2017; Morikawa et al., 2017). Both PNN and ECM formation impairments are associated with neurodegenerative and psychiatric disorders including epilepsy, Alzheimer’s disease and schizophrenia (Heck et al., 2004; Pitkänen et al., 2014; Dzyubenko et al., 2016; Song and Dityatev, 2018). Hence, it is of importance to study not only how factors released by cells impact the ECM, but also how molecules and receptors located at the plasma membrane, Lrp1 included, interact with the ECM and modify its composition.

## Lrp1 and Lipid Rafts

Lipid rafts are regions of the plasma membrane enriched in cholesterol and sphingolipids that are involved in assembling protein complexes for cell signaling events (Brown and London, 1998, 2000; Simons and Toomre, 2000). In the CNS, lipid rafts are essential for synaptic integrity and they are implicated in the pathogenesis of neurodegenerative diseases (Hicks et al., 2012; Rushworth et al., 2013). Although Lrp1 has been firstly found to localize nearly exclusively to coated pits in, for example, vascular smooth muscle cells (Weaver et al., 1996), Lrp1 localization to caveolae, a specialized type of lipid rafts, was later shown by Boucher et al. (2002) on the example of human fibroblasts. In neurons and neuronal-like PC12 and N2a cells, Lrp1 localizes, at least partially, to lipid rafts (Laudati et al., 2016). Upon the disruption of lipid rafts by methyl-β-cyclodextrin, Lrp1-mediated signaling is impaired, while ligand binding and endocytosis capacities remain intact (Laudati et al., 2016). The authors suggest that the presence of Lrp1 in lipid rafts in neuronal cells is due to the scaffolding activity of PSD-95. PSD-95 is a constituent protein of the post-synaptic complex in excitatory synapses (El-Husseini et al., 2000) and functions in stabilization of dendritic spines (Ehrlich et al., 2007). Lrp1, similarly, is present at the synapse and is known to interact with PSD-95 and to affect NMDAR functioning, long-term potentiation (LTP) and synaptic signaling (May et al., 2004; Liu et al., 2010; Nakajima et al., 2013). A decrease in PSD-95 levels upon Lrp1 deletion has been previously reported for the hippocampi of the α CAMKII-Cre-Lrp1 KO mice (Liu et al., 2010). Simultaneous application of methyl-β-cyclodextrin with either enzymatically inactive tissue plasminogen activator (tPA) or active α-2-M to both PC12 cells and cultured cerebellar granule neurons, blocks neurite outgrowth, as does RAP application (Laudati et al., 2016). The mechanism behind this effect has been traced to the fact that, in PC12 and N2a cells, upon treatment with these ligands, Lrp1 forms a complex with PSD-95 and NMDARs that activates tyrosine receptor kinase receptors, stimulates ERK1/2 activity and leads to various cellular responses, including neurite outgrowth (Bacskai et al., 2000; Mantuano et al., 2013). Interestingly, upon Lrp1 loss or blockage, a reduction in the formation, length, branching and outgrowth of neurites is found (Qiu et al., 2004; Nakajima et al., 2013; Safina et al., 2016). Lrp1 is found essential also for neurite growth inhibition mediated by myelin associated-glycoprotein and CNS myelin (Stiles et al., 2013) and has been found to mediate myelin phagocytosis itself (Gaultier et al., 2009).

**FIGURE 2:**
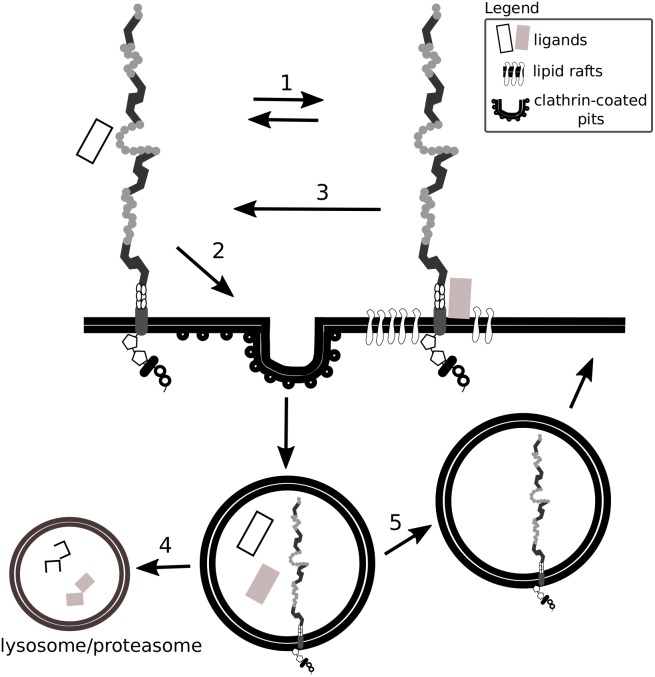
Lrp1 interacts with ligands located in lipid rafts and clathrin-coated pits. Lrp1 distribution in the cell membrane is dynamic: Lrp1 can be found in both lipid raft-containing and lipid raft-free membrane compartments (1). Lrp1-mediated endocytosis requires clathrin and is restricted to lipid raft-free regions (2). Lrp1 can interact with lipid raft-associated receptors and proteins, translocate back to lipid raft-free compartments and mediate the endocytosis of the bound ligand (3). The ligand undergoes either lysosomal or proteasomal degradation (4) while Lrp1 is recycled back to the membrane (5).

By a comparison of various cell types, Wu and Gonias (2005) proposed that the presence of Lrp1 in different plasma membrane compartments depends on the cell type analyzed, as does Lrp1-mediated signaling. For example, although in neuronal and neuronal-like cells Lrp1 is found both in lipid rafts and lipid raft-free membrane compartments, in vascular smooth muscle cells and CHO-K1 cells Lrp1 is present mostly in lipid raft-free membrane compartments (Wu and Gonias, 2005). Lrp1 distribution in the cell membrane remains nevertheless dynamic, as the receptor can translocate from lipid-rafts to clathrin-coated pits where it undergoes endocytosis (Boucher et al., 2002; Wu and Gonias, 2005) (Figure 2). The capacity of Lrp1 to shift between membrane compartments is influenced by plasma membrane microdomains and depends on available extracellular ligands. For example, insulin promotes Lrp1 localization to caveolae in mouse fibroblasts and adipocytes while platelet-derived growth factor (PDGF)-BB decreases it (Zhang et al., 2004). As shown in the example of cultured hepatic cells and mouse embryonic fibroblasts (MEFs), endocytosis of Lrp1 can also occur in lipid rafts. In this case, the kinesin-3 family motor protein, KIF3B, promotes caveolin-dependent endocytosis of Lrp1 by forming a complex with Lrp1 and utrophin, a cytoskeleton protein vital for adequate muscle functioning (Zhang et al., 2004; Kanai et al., 2014).

In the CNS, the localization of Lrp1 to both lipid rafts and clathrin-coated pits has been proposed to be one of the mechanisms enabling a separation of Lrp1 endocytic and signaling activities and their independent regulation (Laudati et al., 2016). For example, PDGF-BB via the PDGF receptor (PDGFR) β and the Src is required for mediating the phosphorylation of tyrosine residues in the cytoplasmic tail of Lrp1 (Boucher et al., 2002; Loukinova et al., 2002). This interaction occurs only when Lrp1 is located to lipid rafts and therefore modulates Lrp1-dependent cell signaling in specific cell compartments (Boucher et al., 2002).

Taken together, interactions between Lrp1, the plasma membrane and ECM molecules highlight the presence of a heterogeneous modulation of Lrp1-dependent cell signaling in specific cell compartments and cell types.

## Lrp1 Interactions With ECM Molecules

Given the multitude of Lrp1 ligands, it is not surprising that ECM molecules also interact with Lrp1. Lrp1 is primarily responsible for the endocytosis and subsequent transport of extracellular proteins to lysosomes (Herz and Strickland, 2001). Although Lrp1-mediated endocytosis occurs in non-lipid raft areas via clathrin-coated pits, due to the receptors’ mobility, GPI proteins like the prion protein and other proteins associated with lipid rafts can also undergo Lrp1-clathrin-dependent endocytosis (Czekay et al., 2001; Wu and Gonias, 2005; Taylor and Hooper, 2007; Jen et al., 2010). Protein complexes and membrane-associated receptors are also Lrp1 ligands. With their endocytosis, Lrp1 further impacts on the protein composition of the plasma membrane and the ECM (Strickland et al., 2002; Gonias et al., 2004).

Although Lrp1’s main function remains the endocytosis of extracellular ligands, it regulates the composition of the ECM also by controlling messenger ribonucleic acid (mRNA) expression and stability (Gaultier et al., 2006). In this way Lrp1 balances the protein levels of, for example, type III collagen, pigment epithelium-derived factor and urokinase-type plasminogen activator (uPA) receptor (uPAR)/Endo-180 in MEFs (Gaultier et al., 2006).

### Lrp1 and HSPGs

Heparan sulfate proteoglycans are core constituents of the ECM implemented in ECM integrity and in facilitating the entry of molecules including morphogens, growth factors and viruses. Some protein ligands of Lrp1 are shown to bind with low affinity to glycosaminoglycan chains of surface HSPGs that in turn facilitate binding to and Lrp1-mediated endocytosis of these proteins (Wilsie and Orlando, 2003; Kanekiyo et al., 2011). Consistent with such a mechanism, the endocytosis of such proteins is blocked by heparin and heparitinase and does not occur in cells lacking HSPGs (Kanekiyo et al., 2011). HSPGs can be cleaved by heparanase-1, an enzyme that requires a proteolytic cleavage to become active. This process is partly dependent on Lrp1-mediated internalization of the inactive pro-enzyme (Vreys et al., 2005). Interestingly, both Lrp1 and HSPGs are required for the endocytosis of mature heparanase-1 (Vreys and David, 2007).

**FIGURE 3:**
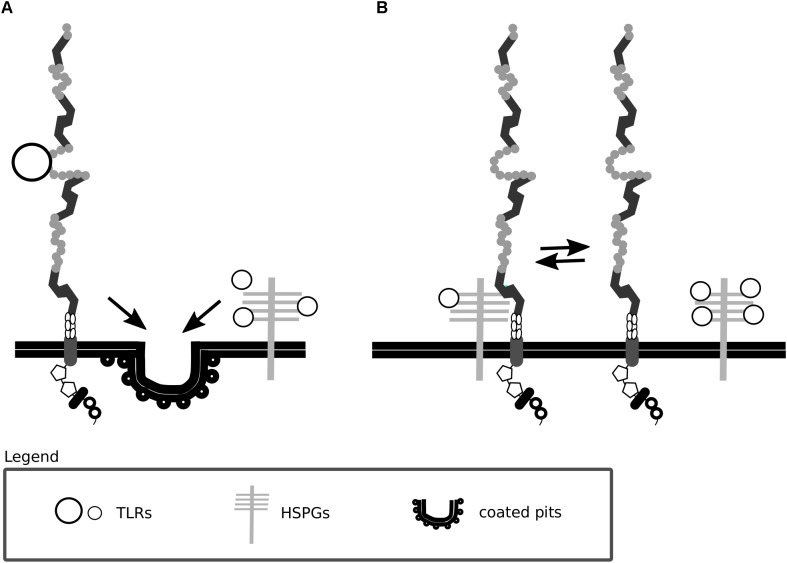
Lrp1 interacts with heparan sulfate proteoglycans. **(A)** Lrp1 and heparan sulfate proteoglycans (HSPGs) are responsible for the independent endocytosis of distinct trigliceride-rich lipoproteins (TRLs) in the liver. **(B)** Lrp1 association with HSPGs determines the availability of lipoprotein binding sites, such that upon the dissociation of Lrp1 from HSPGs, more binding sites appear.

**FIGURE 4:**
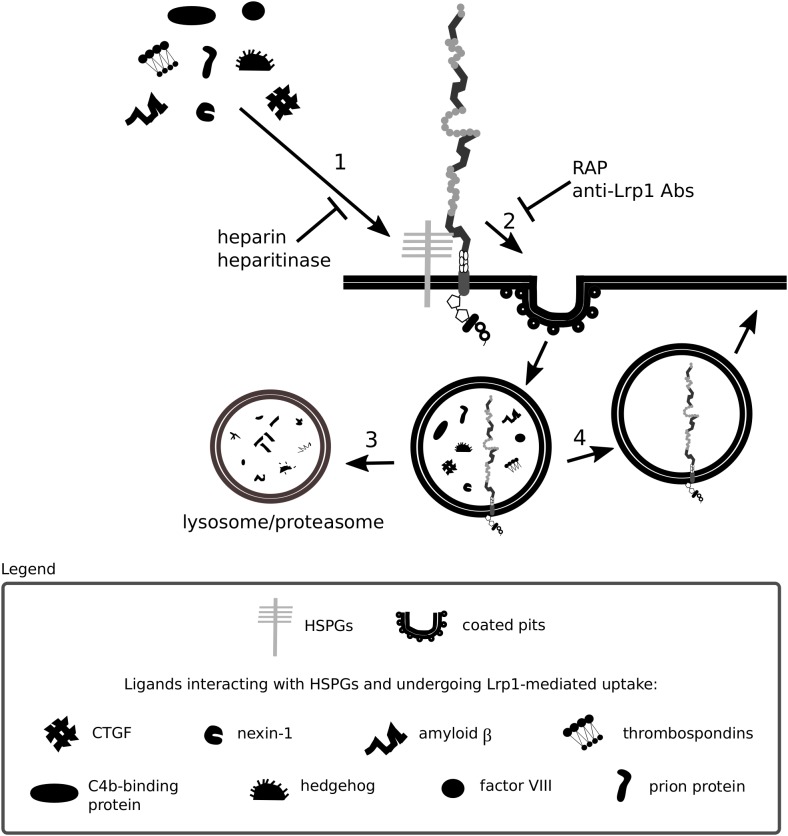
Heparan sulfate proteoglycans act as bridging molecules. Various ligands, including connective tissue growth factor (CTGF) and the prion protein, have been shown to bind to heparan sulfate proteoglycans (HSPGs) (1). After association with HSPGs, the binding of the ligand to Lrp1 is facilitated and Lrp1-mediated endocytosis is triggered (2). Upon endocytosis, the molecules are transported to lysosomes/proteasomes for degradation (3) and Lrp1 is recycled back to the membrane (4). The depicted processes are inhibited both by blockage of Lrp1 and HSPGs.

A summary of Lrp1 interactions with the HSPGs described in this section is presented in Figure 3, 4.

In the liver, Lrp1 was previously shown to clear trigliceride-rich lipoproteins either independently or as a coreceptor with HSPGs (Mahley and Huang, 2007). A recent study questioned this idea and suggested instead that Lrp1 and HSPGs are rather responsible for independent clearing of distinct trigliceride-rich lipoproteins (Foley et al., 2013).

Lrp1 has been proposed to regulate the availability of lipoprotein binding sites by associating with HSPGs. Upon Lrp1 dissociation from HSPGs, more lipoprotein binding sites are revealed, and lipoprotein particle clearance becomes enhanced (Wilsie and Orlando, 2003). These findings were among the first to imply a role for Lrp1 firstly as a mediator of various signaling pathways, independent of its endocytic function and secondly as a regulator of HSPG function. HSPGs were also proposed by the same study to play an active role in lipoprotein uptake (Wilsie and Orlando, 2003). So far, Lrp1 and HSPGs have been shown to mediate the uptake of a complement inhibitor, the C4b-binding protein (Spijkers et al., 2008), the coagulation factor VIII (Sarafanov et al., 2001), cellular prion protein (Sunyach et al., 2003; Taylor and Hooper, 2007), connective tissue growth factor (CTGF) (Gao and Brigstock, 2003) and thrombospondin (Mikhailenko et al., 1995). HSPGs are furthermore found essential for the binding of protease Nexin-1 to Lrp1 (Li X. et al., 2006). Although the internalization of complexed (e.g., with tPA and uPA) and free Nexin-1 is mediated by Lrp1 (Knauer et al., 1997; Crisp et al., 2000), it can be partially substituted by an Lrp1-independent pathway. In Lrp1 deficient cells, the HSPG syndecan-1 takes over the internalization of free Nexin-1 and results in the activation of the Ras-ERK pathway instead of the PKA pathway that becomes active upon Lrp1-mediated internalization (Li X. et al., 2006). Given that Nexin-1 is involved in the regulation of extracellular proteolytic activity, the efficiency of its uptake and cellular responses triggered upon its internalization are crucial, especially for tumor cell invasiveness studies (Li X. et al., 2006).

Another HSPG member involved in Lrp1-mediated endocytosis is glypican-3. Glypican-3 has been shown to regulate embryonic growth by inhibiting the Hedgehog signaling pathway (Capurro et al., 2008). The binding of Hedgehog to glypican-3 triggers Hedgehog:glypican-3 complex endocytosis and degradation, impacting Hedgehog availability for binding with Patched (Capurro et al., 2008). Mechanistically, although glypican-3 and Sonic Hedgehog can directly bind to Lrp1, endocytosis is suggested to occur only upon simultaneous glypican-3 and Sonic Hedgehog interaction with Lrp1 (Capurro et al., 2012). This mechanism is found in MEFs and breast cancer cells, indicating a general role for Lrp1 and HSPGs in Hedgehog signaling (Capurro et al., 2012).

### Lrp1 and Amyloid β

In neuronal cells, APP processing pathways are heterogeneous. In the non-amyloidogenic pathway, APP undergoes an α-and γ-secretase-mediated cleavage that leads to the generation of the soluble α APP fragment and APP C-terminal fragments. When APP is processed in this way, no amyloid β is generated. In contrast, in the amyloidogenic pathway, upon APP cleavage by the β-secretase BACE1 and γ-secretases, amyloid β is produced together with the soluble β APP and APP C-terminal fragments (Chow et al., 2010). Depending on the position of the cleavage, two principal forms of amyloid β can be generated: amyloid β with 40 amino acid residues and amyloid β with 42 amino acid residues. Both forms of amyloid β are released outside the cell in response to normal synaptic signaling and are incorporated into the ECM (Kamenetz et al., 2003; Cirrito et al., 2005). The majority of produced amyloid β is the shorter form. If the longer amyloid β form is produced, it exhibits a higher tendency to form fibrils and later on amyloid plaques that fail to be cleared and therefore accumulate in the brain parenchyma. Amyloid β overproduction, increased processing, aggregation and deposition in the form of plaques leads to neurotoxicity and neurodegeneration and is considered central for Alzheimer’s disease development. It should be noted that, together with amyloid β, various ECM proteins are found in plaques and neurofibrillary tangles, for example ECM-located and cell-surface HSPGs (van Horssen et al., 2003). In this respect, ECM molecules have been analyzed and grouped with regard to the amyloid β form they interact with by Salza et al. (2017). The proteins investigated in this study included reelin, integrins, laminins and collagens. These authors and others showed that fibrillar and oligomeric forms of amyloid β can diversely interact with ECM and membrane proteins, influencing cell homeostasis (Genedani et al., 2010; de Jager et al., 2013; Salza et al., 2017).

For the CNS, the importance of amyloid β generation and its multiplex interactions with the ECM are highlighted by studies showing that the ECM regulates APP levels in fibroblasts and neuronal cells (Bronfman et al., 1996), amyloid β induces ECM degradation in rat astrocytes (Deb et al., 2003) and that both fibrillar and oligomeric forms of amyloid β associate with proteins present at synapses, a characteristic closely related to synapse loss and neurodegeneration observed in Alzheimer’s disease (Selkoe, 2000; Spires-Jones and Hyman, 2014).

**FIGURE 5:**
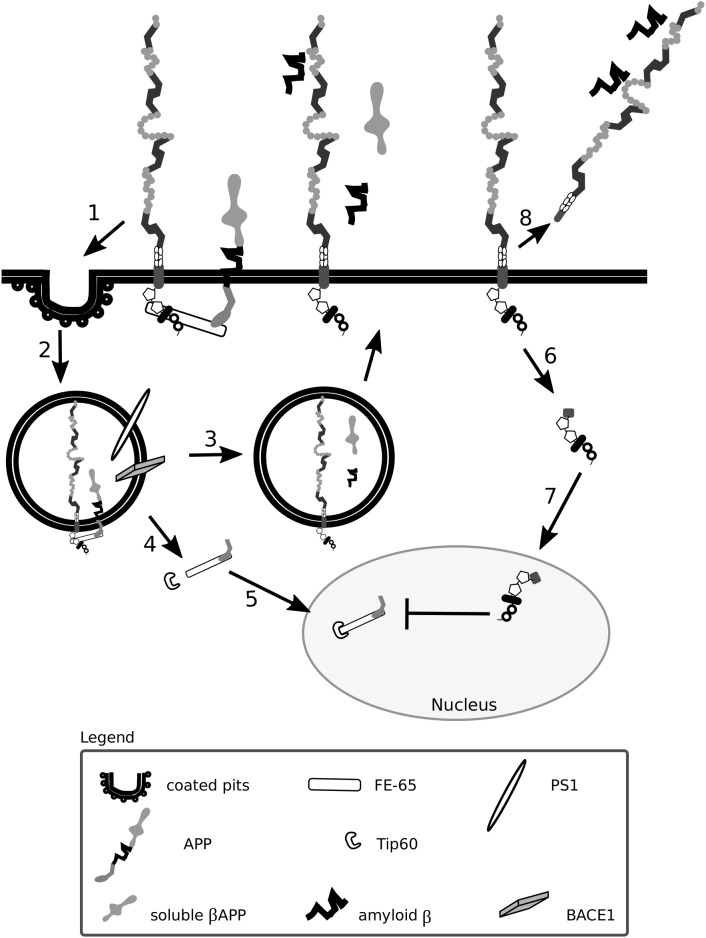
Lrp1 is crucial for the uptake and processing of amyloid precursor protein. The cytoplasmic tail of Lrp1 interacts with the cytoplasmic tail of amyloid precursor protein (APP) via the bridging molecule FE-65. This results in the endocytosis of the Lrp1:APP:FE-65 complex (1) and subsequent proteolysis of APP by the enzyme BACE1 and proteinase presenilin-1 (PS1), which is part of the γ-secretase complex (2). The proteolysis results in the generation of soluble β APP and amyloid β peptides that are released outside the cell in recycling vesicles, like Lrp1 (3). The cytoplasmic tail of APP, after γ-secretase cleavage, forms a complex with FE-65 that interacts with the transcriptional modulator Tip60 (4). This complex translocates to the nucleus where it suppresses the transcription of the Lrp1 promoter, influencing Lrp1-mediated processes (5). Upon γ-secretase-mediated cleavage of Lrp1 (6), its cytoplasmic tail can influence APP processing by competing with the intracellular domain of APP complexed with FE-65 for binding with Tip60 in the nucleus (7). After β-secretase cleavage, the shed extracellular domain of Lrp1 can bind to amyloid β peptides and enhance their clearance (8).

In the CNS, Lrp1 interacts with soluble and transmembrane forms of APP and impacts their internalization, processing and transactivation (Kounnas et al., 1995a; Knauer et al., 1996; Pietrzik et al., 2002) (Figure 5). Upon Lrp1 loss in fibroblasts and CHO cells, APP degradation and trafficking is impaired (Pietrzik et al., 2002). An introduction of a truncated, intracellular domain of Lrp1 to these cells is sufficient to restore APP processing and amyloid β production (Pietrzik et al., 2002). Furthermore, the presence of a functional Lrp1 on the cell surface was shown to reduce the amount of soluble α APP produced and to favor amyloidogenic processing of APP (Ulery et al., 2000).

As Lrp1 and APP are both substrates for γ- and β-secretases, Lrp1 not only stimulates but also interferes with the processing of APP (May et al., 2002; Lleo et al., 2005; von Arnim et al., 2005). APP cleavage is favored when both Lrp1 and APP are present; however, upon Lrp1 overexpression, the cleavage of APP becomes impaired because Lrp1 competes with APP for binding to BACE1 (Lleo et al., 2005; von Arnim et al., 2005; von Einem et al., 2010). Upon γ-secretase-mediated cleavage of Lrp1 (May et al., 2002), the intracellular domain translocates to the nucleus and impacts APP-mediated signaling by interacting with the transcriptional modulator, Tip60 (May et al., 2002; Kinoshita et al., 2003). Lrp1 expression is regulated by APP itself as the APP:FE-65:Tip60 complex is able to suppress the transcription of the Lrp1 promoter (Liu et al., 2007). As a consequence, in fibroblasts of APP knock-out mice Lrp1 expression is increased (Liu et al., 2007). Interestingly, increased levels of APP can lead to a decreased production of shed Lrp1, impairing amyloid β clearance (von Einem et al., 2010).

**FIGURE 6:**
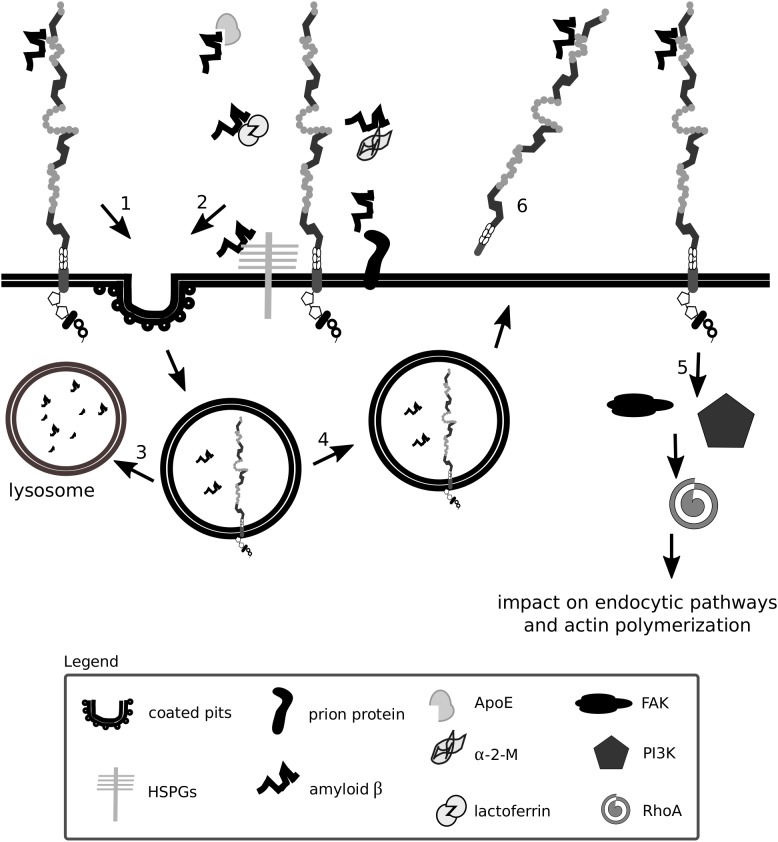
Lrp1 is a receptor mediating amyloid β uptake. Lrp1 can regulate the internalization of amyloid β in several ways. Amyloid β can directly interact with ligand-binding domains of Lrp1 (1). Amyloid β can interact with several molecules including α-2-macroglobulin (α-2-M), apolipoprotein E (ApoE) and heparan sulfate proteoglycans (HSPGs), which bridge it to Lrp1 and facilitate its endocytosis (2). Upon endocytosis, amyloid β can either undergo degradation (3) or, to a smaller extent, be recycled back to the extracellular space (4). Lrp1 can also influence signaling pathways, especially the RhoA pathway that can facilitate amyloid β clearance by stimulating actin polymerization (5). The shed extracellular domain of Lrp1 additionally sequesters soluble amyloid β present at the blood-brain barrier and in the circulation (6).

Fibrils of amyloid β do not accumulate in excessive amounts in healthy individuals due to several efficient clearance mechanisms: extracellular proteolysis, BBB transport, efflux of soluble amyloid β to the peripheral circulation and receptor-mediated endocytosis (Deane et al., 2009; Ramanathan et al., 2015). Lrp1 is vital for the production, internalization and catabolism of amyloid β (Kounnas et al., 1995a; Knauer et al., 1996; Pietrzik et al., 2002; Deane et al., 2004, 2008; Lillis et al., 2008) (Figure 5, 6), and in the brains of Alzheimer’s disease patients, according to some studies, Lrp1 levels are significantly reduced (Kang et al., 1997, 2000; Jaeger and Pietrzik, 2008; Shinohara et al., 2017).

Amyloid β has so far been shown to form complexes with ApoE, lactoferrin, prion protein and activated α-2-M that undergo Lrp1-mediated endocytosis (Qiu et al., 1999; Yang et al., 1999; Kang et al., 2000; Laurén et al., 2009; Rushworth et al., 2013). Monomeric forms of amyloid β can also bind directly to the extracellular ligand-binding domains of Lrp1 and undergo endocytosis (Deane et al., 2004). Microglia were shown to migrate to plaques and engulf amyloid β predominantly via micropinocytosis (Frackowiak et al., 1992; Mandrekar et al., 2009; Lee and Landreth, 2010). Lrp1-mediated amyloid β clearance in pericytes helps prevent amyloid β deposition in the cerebrovasculature (Sagare et al., 2013). Lrp1 expressed on the surface of endothelial cells at the BBB mediates rapid clearance of amyloid β from the brain parenchyma via transcytosis and degradation (Nazer et al., 2008; Yamada et al., 2008; Pflanzner et al., 2011). In the periphery, β-secretase cleaved, shed, soluble Lrp1 can bind amyloid β, further enhancing its clearance from the brain (Sagare et al., 2007). Supporting amyloid β removal via Lrp1 from the system are the liver, spleen, and kidney (Ramanathan et al., 2015). Lrp1 impacts amyloid β metabolism also by activating signaling pathways. By modulating RhoA signaling in Schwann cells, Lrp1 influences cell adhesion and migration (Mantuano et al., 2010). RhoA has been shown to facilitate dynamin-dependent amyloid β endocytosis in neuronal cells (Yu et al., 2010). It is thereby proposed that Lrp1 could activate RhoA and facilitate RhoA-dependent endocytosis of amyloid β (Kanekiyo and Bu, 2014). HSPGs have been identified to be required for the binding of amyloid β and act as a coreceptor for Lrp1 in the process of neuronal amyloid β uptake (Kanekiyo et al., 2011, 2013; Kanekiyo and Bu, 2014). Astrocytic Lrp1 has also been shown to be essential for the uptake and degradation of amyloid β (Kanekiyo and Bu, 2014; Liu et al., 2017). Whether Lrp1 requires HSPGs for amyloid β uptake also in this cell type remains to be elucidated, however, it is suggested that astrocytic Lrp1 affects the degradation of amyloid β in the ECM by modulating the levels of matrix metalloprotease (MMP)-2 and MMP-9 (Yin et al., 2006; Liu et al., 2017).

In summary, although the involvement of Lrp1 in APP and amyloid β processing and Alzheimer’s disease pathogenesis is undeniable, given the complexity of interactions, their true nature may be hidden by the variability in cell lines and the systems used, and is still yet to be fully unraveled.

### Impact of Lrp1 on Cell Adhesion, Migration and Cell Signaling

The ECM is vital for many cellular processes including cell proliferation, survival, differentiation and migration. In order for the cell to migrate, the ECM surrounding it needs to be degraded. This is performed by a variety of proteases including MMPs and serine proteases and tightly controlled by their inhibitors. Both uPA and tPA proteases mediate for example the activation of plasminogen to plasmin that leads to the digestion of the ECM and MMP activation (Werb et al., 1977; Mignatti and Rifkin, 1993; Sappino et al., 1993). One of the first reports showing Lrp1 involvement in ECM turnover and cell migration was published by Okada et al. (1996). Here, by using a Transwell filter migration assay, the authors discovered that the presence of Lrp1 facilitates the migration of smooth muscle cells on fibronectin-coated filters. These observations were reproduced by Wijnberg et al. (1997). The stimulating effect of Lrp1 was not apparent when smooth muscle cells were tested on a collagen gel invasion assay, indicating that the effects of Lrp1 depend on the composition of the matrix (Okada et al., 1996). The addition of uPA, tPA or α -2-M—potent Lrp1 ligands—to the cells is found to be stimulatory for cell migration, neurite outgrowth and axon elongation (Friedman and Seeds, 1995; Holtzman et al., 1995; Okada et al., 1996; Seeds et al., 1996, 1999; Qiu et al., 2004; Lillis et al., 2005).

Lrp1 directly interacts with uPA and the plasminogen activator inhibitor-1 (PAI-1). As the binding affinity of PAI-1 and uPA alone is lower than when in a complex with the uPAR, Lrp1-mediated endocytosis occurs only after PAI-1 binds to the uPA:uPAR complex (Herz et al., 1992; Nykjaer et al., 1994; Nykjaer et al., 1997; Czekay et al., 2001). By the binding and subsequent endocytosis of such complexes, Lrp1 reduces uPAR levels on the cell surface. In this manner, Lrp1 regulates uPAR-mediated proteolysis of the ECM (Conese et al., 1995; Weaver et al., 1996; Nykjaer et al., 1997; Degryse et al., 2001) and impacts plasminogen conversion to plasmin (Weaver et al., 1997). By regulating uPAR levels on the cell surface and decreasing uPA-mediated plasminogen activation, Lrp1 inhibits fibronectin remodeling (Gaultier et al., 2010). By regulating uPAR levels, Lrp1 also influences plasmin-mediated cleavage of ECM glycoproteins lacking collagen in their composition, as well as plasmin-dependent activation of MMP-2 and MMP-9 that degrade the ECM by cleaving collagens (Mignatti and Rifkin, 1993; Brinckerhoff and Matrisian, 2002).

Upon Lrp1 silencing from MEFs, collagen remodeling is increased (Gaultier et al., 2010). This occurs independently of the membrane type-1 matrix metalloprotease (MT1-MMP), a metalloprotease previously shown to impact collagen remodeling and Lrp1 cleavage in malignant cells (Rozanov et al., 2004; Sabeh et al., 2004; Lehti et al., 2009; Sabeh et al., 2009). As upon treatment of MT1-MMP-deficient and wild type skin fibroblasts with RAP collagen remodeling becomes enhanced in both cell types, it hints at the presence of an MT1-MMP-independent pathway that becomes active upon Lrp1 signal blockade.

PAI-1 facilitated migration in rat smooth muscle cells is Lrp1-dependent (Degryse et al., 2004). Interaction of the extracellular heat shock protein 90 with Lrp1 activates Akt 1/2 kinases that facilitate the migration of skin cells to the wound and promote its closure (Tsen et al., 2013). Lrp1 was shown to interact with PDGFR β and regulate PDGFR β cell surface levels and PDGFR β signaling which is of importance, among others, for the integrity of vascular walls and cell chemotaxis (Boucher et al., 2002, 2003; May et al., 2005; Craig et al., 2013; Strickland et al., 2014).

Actin organization and cell migration in smooth muscle cells are, for example, controlled by PDGFR β phosphatidylinositol 3-kinase (PI3K) activation that is Lrp1-dependent (Zhou et al., 2009). The interaction of PDGF β with Lrp1 can also result in the activation of the mitogen-activated protein kinase (MAPK) signaling cascade—a major player in cell survival and proliferation (Takayama et al., 2005).

Mouse embryonic fibroblasts respond with migratory behavior on fibronectin and vitronectin matrixes upon interaction of thrombospondin 1 and the calreticulin:Lrp1 complex (Orr et al., 2003a). The impaired cell migration observed upon Lrp1 loss in MEFs was suggested by Orr et al. (2003a) to be a result of improper lamellipodia generation in Lrp1-deficient cells.

Cell migration is also regulated by Lrp1 via blockage of the effect of stimulatory ligands. This is the case for ApoE, which has been shown to inhibit PDGF-B-dependent smooth muscle cell migration by binding to Lrp1 (Swertfeger et al., 2002). This interaction results in the activation of protein kinase A and increase in intracellular cAMP levels (Swertfeger et al., 2002; Zhu and Hui, 2003) and is of particular importance for protection against vascular disease. ApoE-deficient mice exhibit atherosclerosis (Plump et al., 1992; Zhang et al., 1994), while Lrp1 loss from smooth muscle cells enhances vascular cell activation and also leads to atherosclerosis (Boucher et al., 2003).

Weaver et al. (1997) detected moreover that MEFs that lacked Lrp1 and were plated on serum-, vitronectin-, and fibronectin-coated plates migrated faster than wild type MEFs upon subjection to a scratch assay in the presence of PDGF-BB. This effect was not detectable on Matrigel- and type I collagen-plated cells, highlighting the varying effects of Lrp1 in different matrices. The increased migration capacities of MEFs lacking Lrp1 were suggested by the study to be a result of increased surface levels of the uPAR in these cells (Weaver et al., 1997). As mentioned earlier, Lrp1 can bind PAI-1:uPA:uPAR complexes and facilitate their endocytosis (Nykjaer et al., 1997). This in turn leads to altered levels of the uPAR on the cell surface (Weaver et al., 1997). When cultured under high serum conditions, fibroblasts lacking Lrp1 show increased levels of Rac, a GTPase vital for lamellipodia formation and cell spreading (Ma et al., 2002). In the Lrp1-deficient MEFs, analyzed in the study of Weaver et al. (1997), elevated migration is proposed to be a result of persistent Rac activity and/or increased proteolysis. This reasoning is in agreement with studies showing that, by interacting with proteases, Lrp1 protects ECM proteins from degradation (Herz and Strickland, 2001; Strickland et al., 2002).

A requirement enabling the cell to migrate is the restructuring or disassembly of integrin-linked focal adhesion complexes. Focal adhesion disassembly is mediated by ECM proteins including thrombospondin 1 and 2. Thrombospondins are large oligomeric ECM proteins that participate in cell-cell and cell-matrix interactions by binding to other ECM molecules, cytokines and cell surface receptors. Thrombospondins are considered vital for the development of the CNS, as a lack of thrombospondins 1/2 impairs astrocyte-mediated synaptogenesis and a lack of thrombospondin 1 impairs neural progenitor proliferation and neuronal differentiation *in vivo* and *in vitro* (Christopherson et al., 2005; Lu and Kipnis, 2010). Thrombospondin 1 has been shown to interact with Lrp1, HSPGs, calreticulin and integrins in various cell types (McKeown-Longo et al., 1984; Mikhailenko et al., 1995, 1997; Merle et al., 1997; Li S. S. et al., 2006; Staniszewska et al., 2007).

**FIGURE 7:**
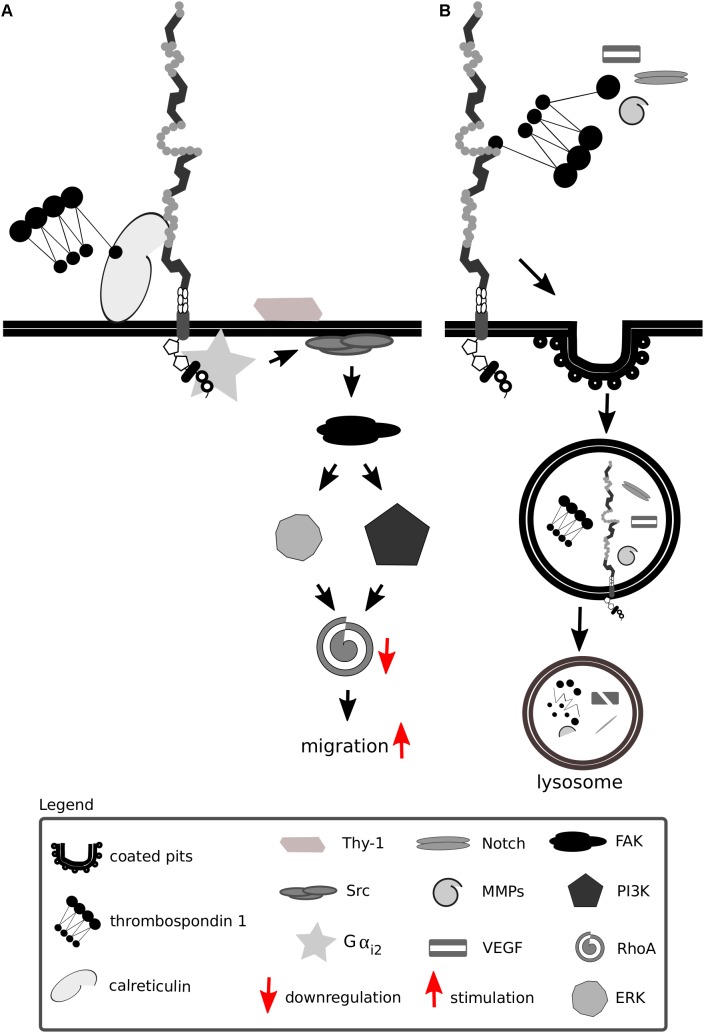
Lrp1 interacts with thrombospondins. **(A)** Upon binding of thrombospondin 1 to calreticulin, its binding to Lrp1 is facilitated. The Lrp1:calreticulin complex leads to the association of the G protein α_i2_ that in turn phosphorylates FAK and Src. Required for the effect of thrombospondin on Src activation is additionally the GPI-linked protein Thy-1. The activation of Src and FAK further activates the ERK and phosphatidylinositol 3-kinase (PI3K) pathways and leads to the downregulation of RhoA, focal adhesion disassembly and cell migration. **(B)** Thrombospondins can function as bridging molecules, enabling Lrp1-mediated endocytosis of various molecules, including Notch, vascular endothelial growth factor (VEGF) and matrix metalloproteinases (MMPs).

Thrombospondins favor cell migration by disassembling and detaching focal adhesions from the ECM—processes dependent on calreticulin and Lrp1 and requiring intact lipid rafts (Orr et al., 2003a,b; Barker et al., 2004; Talme et al., 2013). Both the intact thrombospondin 1 and its cleaved N-terminal domain mediate focal adhesion disassembly (Murphy-Ullrich et al., 1993). The sequence responsible for this effect and binding to calreticulin is located in the N-terminal domain of thrombospondin 1, and a peptide mimetic termed hep I was developed to specifically study interactions of this thrombospondin 1 domain (Murphy-Ullrich et al., 1993). The signaling mediated by thrombospondin 1 via the calreticulin-Lrp1 complex is a process independent of Lrp1-mediated thrombospondin 1 endocytosis (Mikhailenko et al., 1995, 1997) (Figure 7A). Although the sequence responsible for the binding of thrombospondin 1 to Lrp1 and subsequent endocytosis is also located to the N-terminal domain, it does not include the sequence mimicked by hep I, as hep I lacks Lrp1 binding capacity (Orr et al., 2003b; Wang et al., 2004). Interactions of the calreticulin:Lrp1 complex with thrombospondin 1 have been evidenced to result in a temporary association of the G protein α i-2 subunit with Lrp1. This interaction results in FAK and Src phosphorylation (Thy-1-dependent) and activation of ERK, PI3K, and RhoA inactivation and favors cell migration. These events do not occur upon either loss of calreticulin or Lrp1 (Orr et al., 2002, 2003a,b, 2004; Barker et al., 2004).

Thrombospondins also function as bridging molecules between Lrp1 and its extracellular ligands that facilitate their clearance (Figure 7B). Thrombospondin 1 was found to participate in the clearance of vascular endothelial growth factor via Lrp1 in the ovary (Greenaway et al., 2007).

Notch signaling is crucial for proper development, hair pigmentation and homeostasis, and mediates short-range, direct communications between neighboring cells (Schouwey et al., 2007). Lrp1 facilitates Notch 3 *trans*-endocytosis and thrombospondin 2-mediated potentiation of Notch 3 signaling (Meng et al., 2009, 2010), highlighting that the ECM is involved in the modulation of Notch function, and Lrp1 and thrombospondins support non-cell autonomous short-range signaling (Meng et al., 2010).

The activity of MMPs, adamalysin-like metalloproteinases with thrombospondin domains and membrane-anchored adamalysins, is tightly regulated by four members of the tissue inhibitors of the MMPs (TIMPs) family. In the TIMP family, TIMP3 is unique as it controls the activity of all three metalloproteinase classes. The lack of TIMP3 leads to excessive ECM turnover caused by uncontrolled proteinase activity. TIMP3 extracellular levels are regulated by Lrp1-mediated endocytosis (Scilabra et al., 2013; Thevenard et al., 2014). The shed, soluble Lrp1 competes with cell-surface Lrp1 for TIMP binding and results in increased extracellular levels of TIMP, promoting its inhibitory action (Scilabra et al., 2013, 2017).

In addition, Lrp1 mediates the endocytosis of MMP-2, MMP-9, and MMP-13 and adamalysin-like metalloproteinases with thrombospondin domains 4 and 5, preventing their proteolytic and signaling functions (Emonard et al., 2005; Yamamoto et al., 2014). Thrombospondin 1/2 was also evidenced to interact with MMP-2 and MMP-9 (Bein and Simons, 2000). Upon binding of pro-MMP-2 to thrombospondin 2, the complex associates with Lrp1 and undergoes endocytosis, which can be blocked by an anti-thrombospondin 1 antibody (Yang et al., 2001; Emonard et al., 2004).

MMP-2 activity favors angiogenesis and endothelial cell invasion in malignant gliomas. In thrombospondin 2 knock-out mice, microvessel density is enhanced and MMP-2 levels are significantly upregulated (Fears et al., 2005). MMP-2 levels and invasion capabilities of microvessel endothelial cells in these mice are reduced upon addition of thrombospondin 2. This effect is blocked by downregulating Lrp1. Curiously, the endocytosis of pro-MMP-2 can also take place by formation of complexes with TIMP-2 and is Lrp1-dependent. This interaction, however, does not require thrombospondin (Emonard et al., 2004).

Vital for the normal function of elastic arteries are collagens and elastins, prominent members of the ECM. It is appreciated that excess protease activity can contribute to the elastic fiber degradation in these vessels. Smooth muscle cells are essential for establishment of proper vessel diameter, correct deposition of the ECM and assembly of elastic fibers. The deletion of Lrp1 from the embryo proper results in severe impairments in investment of vessels with pericytes and vascular smooth muscle cells. The deletion of Lrp1 from vascular smooth muscle cells leads to increased proliferation and aneurysms (Boucher et al., 2003; Nakajima et al., 2014). The deletion of Lrp1 from smooth muscle cells also impacts the integrity of the vasculature by altering PDGFR β signaling and levels of various ECM molecules pivotal for proper vessel development, contributing to the development of hypertension, atherosclerosis and other cardiovascular diseases (Zhou et al., 2009; Strickland et al., 2014). Alongside a lack of Lrp1 in smooth muscle cells, matrix decomposition becomes dysregulated due to CTGF protein level upregulation (Muratoglu et al., 2013). CTGF accumulation is traced to the missing Lrp1-mediated clearance of CTGF (Segarini et al., 2001). Lrp1-mediated endocytosis of CTGF occurs tissue-independently as it has also been shown to take place in the liver (Gao and Brigstock, 2003; Gerritsen et al., 2016). The disrupted elastin fiber architecture and ECM disorganization upon Lrp1 deletion from smooth muscle cells was found by Muratoglu et al. (2013) to increase protein levels of MMP-2, MMP-9, MT1-MMP, and serine protease high-temperature requirement factor A1 (HtrA1). HtrA1 is abundant in smooth muscle cells, degrades many ECM proteins including aggrecan, fibulin-5 and collagens and has been previously associated with disorganized elastic fibers (Hadfield et al., 2008; Vierkotten et al., 2011). Lrp1 was found by Muratoglu et al. (2013) to regulate the levels of HtrA1 in the vessel wall by rapid endocytosis of this protease, thereby impacting vessel wall integrity.

In conclusion, the presence of different tissue-dependent Lrp1-mediated mechanisms for serine protease and MMP

clearance highlights that Lrp1-mediated uptake and degradation provides a vital mechanism for limiting excessive extracellular proteolytic activity (Yang et al., 2001). By eliciting control over MMP extracellular levels, Lrp1 additionally modifies cell adhesion and migration capabilities that are crucial not only for processes like wound healing and angiogenesis but also for tumor growth (Fears et al., 2005; Van Gool et al., 2015). For example, EGF-mediated downregulation of Lrp1 activity in astrocytic tumors impacts ECM composition and may contribute to tumor invasiveness (Hussaini et al., 1999).

### Lrp1 Binding to and Regulation of Integrins

Integrins are transmembrane glycoprotein receptors consisting of α and β heterodimers. Integrins have been implemented in molecular adhesion, cell survival and migration (Pan et al., 2016).

The process of binding of integrins to the ECM provokes a change in conformation of integrins that connects the ECM with the cell and leads to adhesion. An integrin that is especially crucial for triggering the cell-matrix interactions is β1 integrin. β1 integrin outside-in signaling leads to integrin recruitment and clustering, phosphorylation of focal adhesion kinase and the assembly of focal adhesions, promoting cell spreading and adhesion.

Fibronectin is a ubiquitous and multifunctional ECM protein vital for cell adhesion, migration and differentiation. Lrp1 participates in the catabolism of fibronectin by mediating fibronectin endocytosis (Salicioni et al., 2002). In particular, Lrp1 prevents fibronectin upregulation on the cell surface and in the cell-secreted medium, as shown for MEFs and Chinese Hamster ovary cells (Salicioni et al., 2002).

**FIGURE 8:**
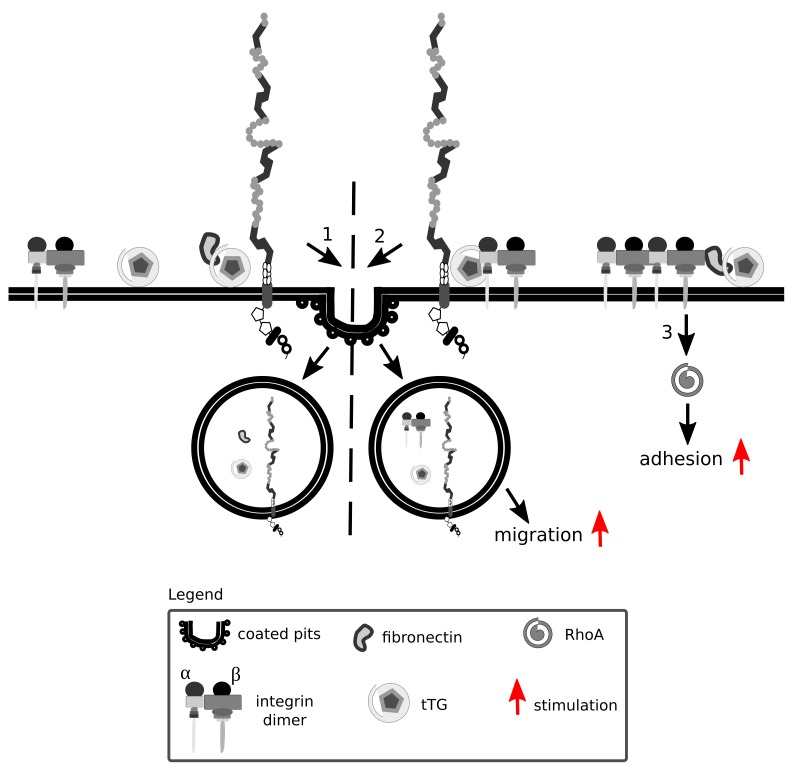
Lrp1 is vital for integrin signaling and function. Integrins are formed by α and β heterodimers. Fibronectin and integrins can interact with tissue transglutaminase (tTG) and impact on cell migration and adhesion. Upon fibronectin binding, surface tTG is endocytosed by Lrp1 (1). tTG mediates the formation of Lrp1:integrin complexes that facilitate integrin endocytosis and cell migration (2). Interaction of tTG with integrins stabilizes fibronectin:integrin complexes and stimulates RhoA activation and cell adhesion (3).

Tissue transglutaminase is a ubiquitously expressed Ca^2+^-dependent protein crosslinking enzyme present in the cytoplasm, at the cell surface and in the ECM. Fibronectin and β integrins are major interaction partners of tissue transglutaminase that impact on cell adhesion and migration (Turner and Lorand, 1989; Akimov et al., 2000) (Figure 8). Fibronectin, aside from being internalized in an Lrp1-dependent manner, also acts as a bridging molecule and enhances Lrp1-mediated surface tissue transglutaminase endocytosis (Zemskov et al., 2007).

Interaction of tissue transglutaminase with β integrins results in the formation of fibronectin-tissue transglutaminase-integrin complexes that stabilize the interactions of fibronectin with integrins (Akimov et al., 2000). The interaction of tissue transglutaminase with integrins facilitates their clustering and activates RhoA (Janiak et al., 2006).

Surface tissue transglutaminase has been recently shown to associate with Lrp1 and promote the formation of β1 integrin-Lrp1 complexes (Zemskov et al., 2007). As tissue transglutaminase associates with integrins, they are internalized by Lrp1 as a complex, resulting in a modification of cell-matrix interactions. Upon Lrp1 loss, transglutaminase activity of surface tissue transglutaminase is upregulated and cell adhesion enhanced (Zemskov et al., 2007).

Fibulin-5 is an ECM integrin-binding protein that is involved in elastic fiber formation (Yanagisawa et al., 2002). The binding of fibulin-5 to uPA stimulates plasminogen activation. This leads to the proteolysis of fibulin-5, its dissociation from integrins and stimulation of β1 integrin-mediated migration of MEFs, independently of the uPAR (Kapustin et al., 2012). Czekay et al. (2003) found, however, that if uPARs are associated with integrins and the uPA:PAI-1 complex binds to such an uPAR, integrins are internalized by Lrp1 together with the uPA:PAI-1:uPAR complex, influencing integrin functioning.

**FIGURE 9:**
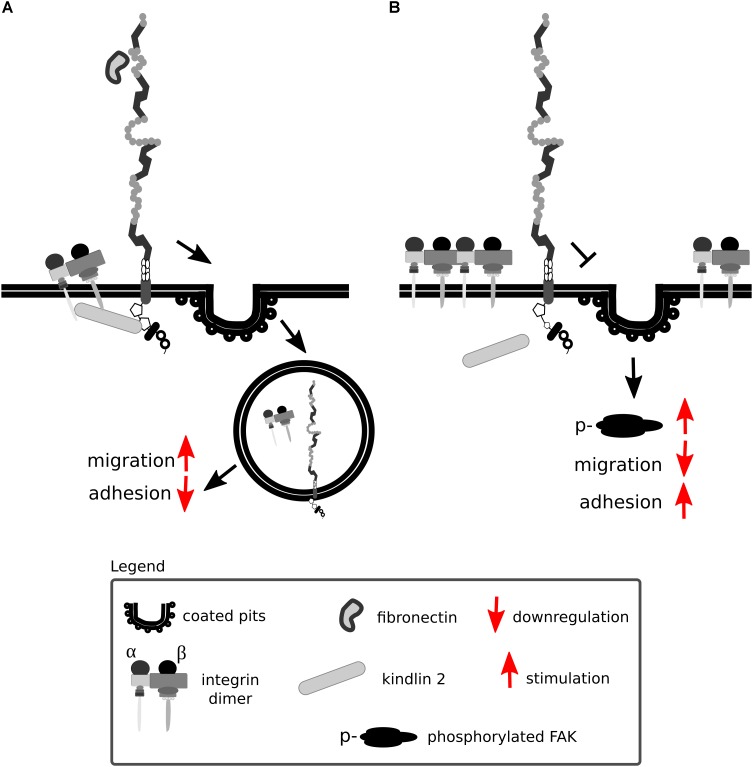
Lrp1 mediates β1 integrin endocytosis. **(A)** The NPxY2 motif located in the cytoplasmic domain of Lrp1 is responsible for the endocytosis of β1 integrins via kindlin 2 that enhances cell migration. **(B)** Upon mutations in the NPxY2 motif of Lrp1, interactions with kindlin 2 do not occur, resulting in reduced β1 integrin recycling and facilitation of cell adhesion.

Lrp1 promotes β1 integrin activation via kindlin 2 and is considered to be a driver for the trafficking and proteasomal and lysosomal degradation of endocytosed activated β1 integrin via the protein kinase C (Wujak et al., 2017) (Figure 9A). Knock-in mutations in the intracellular NPxY2 motif of Lrp1, similarly to the knock-out of Lrp1, lead to elevated levels of immature β1 integrin on the cell surface, disrupting β1 integrin functionality (Salicioni et al., 2004; Rabiej et al., 2015; Wujak et al., 2017). Immature β1 integrin lacks the full glycosylation pattern that is acquired in the endoplasmic apparatus and the Golgi complex. Changes in glycosylation patterns are known to disturb transit through the Golgi apparatus, formation of complexes with α integrin subunits and ligand-binding affinities (Bellis, 2004). Knock-in mutations in the intracellular NPxY2 motif of Lrp1 lead moreover to reduced β1 integrin recycling and cause increased cell adhesion to collagen and fibronectin matrices and reduced cell migration of MEFs (Rabiej et al., 2015). This effect is due to the fact that the impaired Lrp1 endocytosis of β1 integrin leads to increased numbers of focal adhesions, increased focal adhesion kinase phosphorylation status and decreased MMP-2 and MMP-9 activity in MEFs and neurons (Rabiej et al., 2015) (Figure 9B).

Interactions of Lrp1 with β1 integrin are proposed to be similar to Lrp1 interactions with APP. The knock-in mutation in the intracellular NPxY2 motif of Lrp1 leads to surface accumulation of not only β1 integrin but also APP (Pietrzik et al., 2002; Rabiej et al., 2015). β1 integrin and APP are also endocytosed together by Lrp1 upon complex formation with the scaffolding protein Ran-binding protein 9, an interaction of importance for Alzheimer’s disease pathogenesis and synaptic plasticity (Woo et al., 2012a,b).

In the CNS, β1 integrin is known for participating in neuronal migration and neurite outgrowth (Belvindrah et al., 2007b; Ribeiro et al., 2013; Rabiej et al., 2015), however, its function is not limited to cell adhesion and migration. β1 integrin is expressed by NSPCs and is vital for the formation of cell layers in the cerebral cortex (Belvindrah et al., 2007a). An *in vivo* conditional deletion of β1 integrin in radial glia cells leads to spontaneous seizures and reactive astrogliosis (Robel et al., 2009, 2015). β1 integrin presence is put forward as being inhibitory for astrocyte differentiation in adult hippocampal neural stem cells as well as in embryonic neural stem cells (Pan et al., 2014; Brooker et al., 2016). This inhibitory effect is mediated via the integrin-linked kinase (Pan et al., 2014; Brooker et al., 2016) and appears to be more prominent in female mice, as shown by Brooker et al. (2016). Interestingly, integrin-linked kinase, as a major downstream effector kinase of β1 integrin signaling, is vital for ECM deposition and controls its expression.

In a different β1 integrin mutant, β1 integrin loss restricted to astrocytes has been found to impact endothelial cell number at the neurovascular unit, blood vessel branching and aquaporin 4 levels (Venkatesan et al., 2015). Given that Lrp1 is found on astrocytes and contributes to vessel development as well as BBB integrity (Boucher et al., 2003; Nakajima et al., 2014; Fredriksson et al., 2015; Auderset et al., 2016) it is of interest for future studies to elucidate to what extent Lrp1 participates in β1 integrin-mediated effects in the developing CNS.

As Lrp1 was recently identified as a receptor for β1 integrins also in thyroid cancer cells (Theret et al., 2017), Lrp1 and integrins emerge as potential candidate genes for preventing cancer invasiveness.

Lrp1 has also been shown to bind β2 integrins located on leukocytes and monocytes (Spijkers et al., 2005; Cao et al., 2006). The interactions of Lrp1 with β2 integrins are suggested to modulate cell adhesion and migration by regulating integrin recycling, as shown in the example of macrophages (Cao et al., 2006). Hypercholesterolemia is proposed to induce hematopoietic stem/progenitor cell (HSPC) proliferation and their migration into lesioned sites, where they are suggested to undergo differentiation into leukocytes and form plaques found in arteriosclerosis patients (Wang et al., 2014). Vital for HSPC adhesion, migration and homing is β2 integrin (Wang et al., 2014). Loss of Lrp1 does not influence expression levels of β2 integrin, but leads to inhibition of HSPC adhesion, suggesting Lrp1 in this context impacts β2 integrin functioning (Wang et al., 2014).

### Lrp1 Interactions With tPA

**FIGURE 10:**
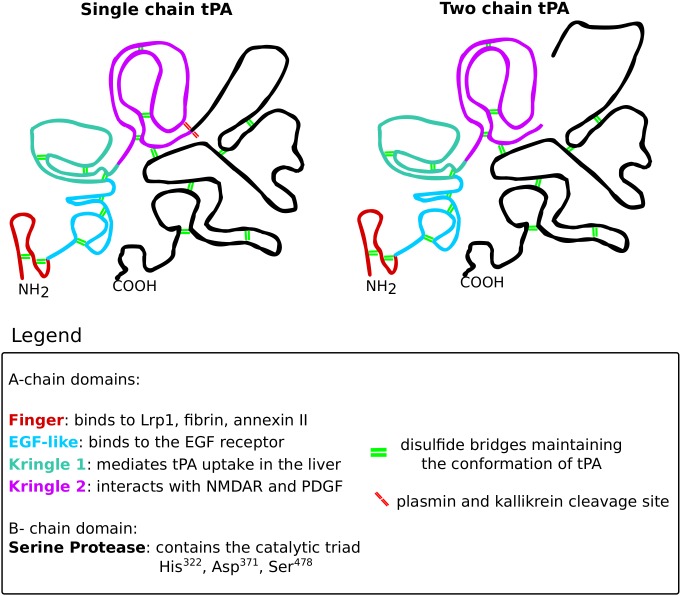
The structure of tissue plasminogen activator. Tissue plasminogen activator (tPA), like all serine proteases, exists in two forms: the single-chain and the two-chain form. The conformation of tPA is maintained by 17 disulfide bridges (green double lines). The structure of both tPA forms consists of two chains: the A-chain and the B-chain. The A-chain consists of four domains that are color-coded on the scheme. The finger domain (red) is responsible for interacting with Lrp1, fibrin and annexin II. The EGF-like domain (blue) binds the EGF receptor. The kringle 1 domain (green) mediates tPA uptake in the liver. The kringle 2 domain (violet) is vital for interactions with NMDARs and PDGFs. The B-chain (black) consists of the catalytic triad that mediates the proteolytic activity of tPA. The single-chain form of tPA is catalytically active and can be further cleaved by kallikrein and plasmin (red double lines), resulting in the two-chain form of tPA. The two-chain tPA is also catalytically active.

Tissue plasminogen activator is a serine protease that belongs to the chymotrypsin family, mostly known for its role in degrading the extracellular matrix components via activating plasmin. This glycoprotein is built up from the heavy A-chain and the light B-chain (Figure 10). The heavy A-chain is composed of the finger domain, EGF-like domain and Kringle 1 and 2 domains. The finger domain is responsible for binding to fibrin and the membrane receptors Annexin II and Lrp1 (Kagitani et al., 1985; Bu et al., 1992; Hajjar et al., 1994). The EGF-like domain is crucial for binding to the EGF receptor (Correa et al., 2011). The Kringle 1 domain is involved in the uptake of tPA in the liver and the Kringle 2 domain is vital for interactions with NMDARs and PDGFs (Kuiper et al., 1988; Fredriksson et al., 2004; Lesept et al., 2016). The light B-chain comprises the serine protease domain with the catalytic triad (His^322^, Asp^371^, and Ser^478^), responsible for the proteolytic activity of tPA.

Unlike other members of the chymotrypsin family, tPA is secreted as a proteolytically active single chain form that can be subsequently processed into the equally proteolytically active two-chain tPA by plasmin or kallikrein. Given its prominent matrix degradation capacities, tPA levels and activity must be strictly regulated. In the CNS, Lrp1 together with protease inhibitors PAI-1 and neuroserpin is especially crucial for controlling the effect of tPA on ECM remodeling (Orth et al., 1992; Makarova et al., 2003; Yepes and Lawrence, 2004; Czekay et al., 2011; Hébert et al., 2015).

**FIGURE 11:**
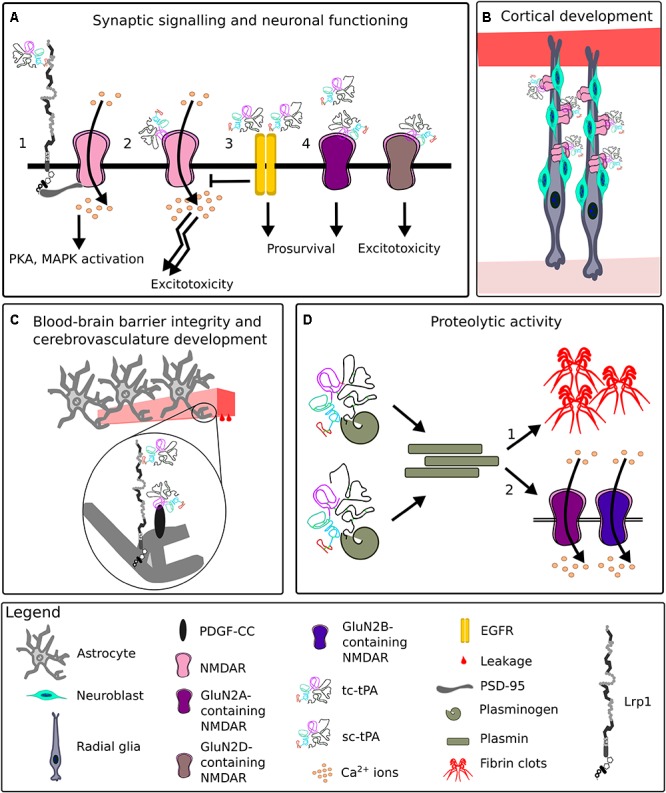
Representative functions of tissue plasminogen activator in the CNS. **(A)** At the synapse, upon binding of tissue plasminogen activator (tPA), Lrp1 recruits postsynaptic density protein 95 (PSD-95). PSD-95 bridges the NPxY2 motif of Lrp1 and *N*-methyl-D-aspartate receptor (NMDAR). NMDAR allows Ca^2+^ influx, leading to an activation of signaling cascades (1). TPA can directly interact with the NMDAR, enhancing its extrasynaptic surface diffusion and resulting in excitotoxicity (2). Interactions of low levels of single-chain tPA (sc-tPA) and two-chain tPA (tc-tPA) with epidermal growth factor receptor (EGFR) result in neuroprotection and inhibition of NMDAR neurotoxicity (3). Interactions of tPA with synaptic GluN2A-NMDARs are neuroprotective, while with extrasynaptic GluN2D-NMDARs, neurotoxic (4). **(B)** During corticogenesis, tPA released by migrating neuroblasts clusters NMDARs on radial glia cells and ensures proper cortical migration and maturation of neurons. **(C)** TPA enhances blood-brain barrier permeability by interacting with astrocytic Lrp1 and platelet-derived growth factor (PDGF)-CC. **(D)** Both sc-tPA and tc-tPA are proteolytically active and convert plasminogen to plasmin. Plasmin digests fibrin, a process especially vital for renewing blood flow after ischemia (1). Plasmin cleaves GluN2A and GluN2B-containing NMDARs, impacting NMDAR functionality (2).

For many years, tPA has been mostly appreciated for its fibrinolytic activity, however, it possesses a plethora of other functions vital for CNS homeostasis, some of which are summarized in Figure 11 (Sappino et al., 1993; Briens et al., 2017; Hébert et al., 2015).

TPA, alike Lrp1, is implicated in the pathology of neurodegenerative diseases, including Alzheimer’s disease, psychotic disorders like schizophrenia (Fabbro and Seeds, 2009; Hoirisch-Clapauch and Nardi, 2015) and is recognized for supporting BBB integrity (Polavarapu et al., 2007; Fredriksson et al., 2015; Stefanitsch et al., 2015).

The tPA has been found to facilitate neurite outgrowth by promoting local proteolysis (Seeds et al., 1996) and to favor neuronal (synaptic) maturation after differentiation from cortical neural progenitor cells (Lee et al., 2014). TPA is highly expressed in brain regions undergoing cellular migration. TPA gene expression is found most prominently in the cerebellum in the period of neuronal migration and is induced in granule neurons leaving the external granule layer of the cerebellum (Friedman and Seeds, 1995). Mice that lack tPA show an increased amount of granule neurons migrating in the molecular layer of the cerebellum (Seeds et al., 1999). The mechanism behind enhanced tPA expression in granule cells is suggested to be related to NMDAR signaling as these receptors are crucial for granule cell migration (Komuro and Rakic, 1992, 1993; Friedman and Seeds, 1995). TPA-mediated signaling is also supportive for Schwann cell migration via direct interaction with NMDARs and is present even upon blockage of Lrp1 (Mantuano et al., 2015). Recently, tPA has been implicated in the control of proper radial glial cell organization and differentiation. The interaction between neuronal tPA and NMDAR on radial glia cells was found to be crucial for proper cortical migration and maturation (Pasquet et al., 2018). In a mouse model of Fragile X syndrome, a stimulating effect of tPA on Fmr1-deficient cells migrating out of neurospheres was also apparent (Achuta et al., 2014).

TPA and plasmin have been shown to regulate mossy fiber outgrowth in the hippocampus via the proteolysis of the proteoglycan DSD-1/phosphacan (Wu et al., 2000).

In murine renal and non-renal myofibroblasts, tPA promotes transforming growth factor (TGF)-β1-mediated activation and ECM production which is independent of its proteolytic activity. TPA exerts this action by directly activating Lrp1 by a phosphorylation of tyrosine residues located in the C-terminus of Lrp1. This process results in Lrp1-mediated β1 integrin recruitment and integrin-linked kinase signaling (Hu et al., 2007). Upon Lrp1 loss, tPA fails to induce myofibroblast activation and ECM overproduction as evidenced by type I collagen expression (Hu et al., 2007). TGF-β1 fibrogenic activity is not disturbed in Lrp1-deficient cells, suggesting Lrp1 loss affects only tPA-mediated signaling (Hu et al., 2007). In other cell types, however, Lrp1 was shown to impact TGF-β expression and signaling, for example during vessel wall remodeling (Muratoglu et al., 2011).

TPA is not only supportive of cell migration, but, under certain conditions, it also favors cell adhesion. This effect is reversed upon binding of tPA to PAI-1, which induces an Lrp1-mediated internalization of the adhesion complex and cell detachment (Cao et al., 2006; Kozlova et al., 2015).

Extracellular matrix remodeling by tPA and other proteases is not only involved in axonal growth and cell migration but also activates cellular signaling cascades. For example, degraded fibronectin particles interact with integrin receptors and trigger cell signaling that further remodels the ECM by regulating mRNA levels of metalloproteases and other factors (Werb et al., 1989). TPA also acts as a cytokine by upregulating MMP-9 gene expression in an Lrp1-dependent manner (Wang et al., 2003; Yepes et al., 2003; Hu et al., 2006). Lrp1 expression is increased upon ischemic stroke, and treatment with RAP or antibodies against Lrp1 decreases the degree of tissue edema after middle cerebral artery occlusion (Polavarapu et al., 2007). Vasocontraction of smooth muscle cells is facilitated by tPA and requires a functional interaction between Lrp1 and α and β integrins (Akkawi et al., 2006). Astrocytic Lrp1 and tPA have been directly shown to regulate BBB permeability (Polavarapu et al., 2007; Su et al., 2008; Niego et al., 2012). Improper tPA-Lrp1 and tPA-Mac-1-Lrp1-PDGFR α interactions at the BBB are known to influence the permeability of the neurovascular unit in relation to seizures and ischemia (Polavarapu et al., 2007; Zhang et al., 2007, 2009; Su et al., 2008; Nakajima et al., 2014; Fredriksson et al., 2015).

In the CNS, tPA is synthesized and released by various cell types including neurons and astrocytes (Hébert et al., 2015). The majority of neuronal tPA is present in dendrites and axons and is released following depolarization of presynaptic terminals. tPA liberation impacts the release of glutamate, thereby exerting a modulatory function on synaptic activity (Wu et al., 2015; Yepes et al., 2016). Simultaneously, tPA release is proposed to exhibit an independent, homeostatic effect on the cortical glutamatergic postsynapse, which is dependent on the degree of neuronal activation and extracellular Ca^2+^ concentrations (Jeanneret et al., 2016).

The tPA interacts with proteins and receptors located at the synapse, among them Lrp1 and NMDAR (Nicole et al., 2001; Pawlak et al., 2005; Benchenane et al., 2007; Macrez et al., 2010; Jullienne et al., 2011; Ng et al., 2012; Obiang et al., 2012; Parcq et al., 2012; Mantuano et al., 2013; Lesept et al., 2016). Both single- and two-chain tPA at low concentrations can activate neuronal EGF receptors, which decrease NMDAR signaling and result in neurotrophic effects. High concentrations of single-chain tPA lead to neurotoxic effects by activating NMDAR signaling (Bertrand et al., 2015; Chevilley et al., 2015). Neuronal survival is enhanced via astrocytic TGF-α induction of PAI-1. PAI-1 inhibits tPA and thereby prevents NMDAR-mediated neurotoxicity (Gabriel et al., 2002). Additionally, tPA exerts neuroprotective effects by activating the annexin II and mTOR pathways (Chevilley et al., 2015; Grummisch et al., 2016; Lemarchand et al., 2016).

The interaction of tPA with Lrp1 is vital for the generation of hippocampal late LTP and enhancement of PKA activity (Zhuo et al., 2000; Makarova et al., 2003).

The tPA is tightly connected with LTP induction and synaptic signaling also by its ability to degrade the ECM (Qian et al., 1993; Huang et al., 1996; Baranes et al., 1998; Madani et al., 1999; Trotter et al., 2014). TPA-mediated proteolytic cleavage of plasminogen results in the generation of plasmin that enhances the NMDAR-mediated increase in Ca^2+^ influx upon glutamate application to cultured hippocampal neurons (Inoue et al., 1994). TPA and plasmin have also been shown to convert pro brain-derived neurotrophic factor to mature brain-derived neurotrophic factor, a protein critical for LTP generation (Pang and Lu, 2004; Pang et al., 2004).

Healthy astrocytes possess the ability to uptake tPA, via Lrp1, from the synaptic cleft and modulate the efficacy of synaptic responses (Makarova et al., 2003; Fernández-Monreal et al., 2004; Cassé et al., 2012). Meanwhile, *in vitro* cultured astrocytes with reduced Lrp1 levels do not uptake neuron-derived tPA efficiently and as a result cause elevated levels of tPA in the synaptic cleft (Cassé et al., 2012). Astrocytic Lrp1 uptake of tPA is thereby crucial in preventing surplus tPA-mediated activation of NMDARs and neuronal cell death (Cassé et al., 2012) and is a promising target for epilepsy research.

The tPA is an immediate early gene expressed not only upon LTP but also early after seizures (Qian et al., 1993; Carroll et al., 1994). The exact impact of tPA on seizure generation and epilepsy progression is, however, complex (Tan et al., 2012). TPA-deficient mice are resistant to excitotoxin-induced neuronal death, but mice overexpressing tPA in adult neurons do not show neurodegeneration, only a selective enhancement of hippocampal LTP and memory (Tsirka et al., 1997; Madani et al., 1999). Involvement of tPA in activity-dependent synaptic plasticity has also been hypothesized to occur in the cerebellum upon motor learning. Here, tPA secreted at either Purkinje cell dendrites or granule neurons and parallel fibers, could eliminate synapses by degrading cell surface receptors and adhesion molecules, or facilitate new synapse formation by degrading the ECM (Seeds et al., 1995, 1996).

Lrp1 and the extracellular protease tPA impact cell migration, adhesion and NMDAR-mediated signaling. TPA exerts its effects not only via its proteolytic activity but also via activating signaling cascades (Nicole et al., 2001; Yuan et al., 2009; Parcq et al., 2012; Chevilley et al., 2015). Interactions of Lrp1 with tPA have been shown so far to depend on the animal age, the cell type, the exact co-factors available, the extracellular/intracellular tPA ratio and the form of tPA. These myriad parameters need consideration when studying the intriguing relationship between Lrp1 and tPA and partially explains contradictory findings in the literature regarding the function of this ECM protein (Nicole et al., 2001; Fernández-Monreal et al., 2004; Benchenane et al., 2007; Jullienne et al., 2011; Robinson et al., 2015).

## Conclusion

Lrp1 is a potent modulator of ECM function in the CNS and beyond. Lrp1 can impact on the composition of the cell plasma membrane, and thereby the ECM, by the endocytosis of a wide range of ECM proteins and protein complexes. ECM molecules themselves can facilitate Lrp1-mediated endocytosis by acting as bridging and docking molecules, as do surface-bound HSPGs. By mediating the endocytosis of a wide range of proteases and protease inhibitors, Lrp1 directly impacts ECM composition. By associating with scaffolding and adaptor proteins and forming co-receptor complexes, Lrp1 appears vital for cell signaling events and activation of many downstream signaling cascades that lead to, among others, ECM remodeling. In this context, the interactions of Lrp1 with its ligands, especially tPA and integrins, have proven critical for normal development, synaptic signaling, cell adhesion and migration. Therefore, it is not surprising that changes in Lrp1 expression and phosphorylation status are associated with neurodegenerative and cardiovascular diseases and cancer.

**FIGURE 12:**
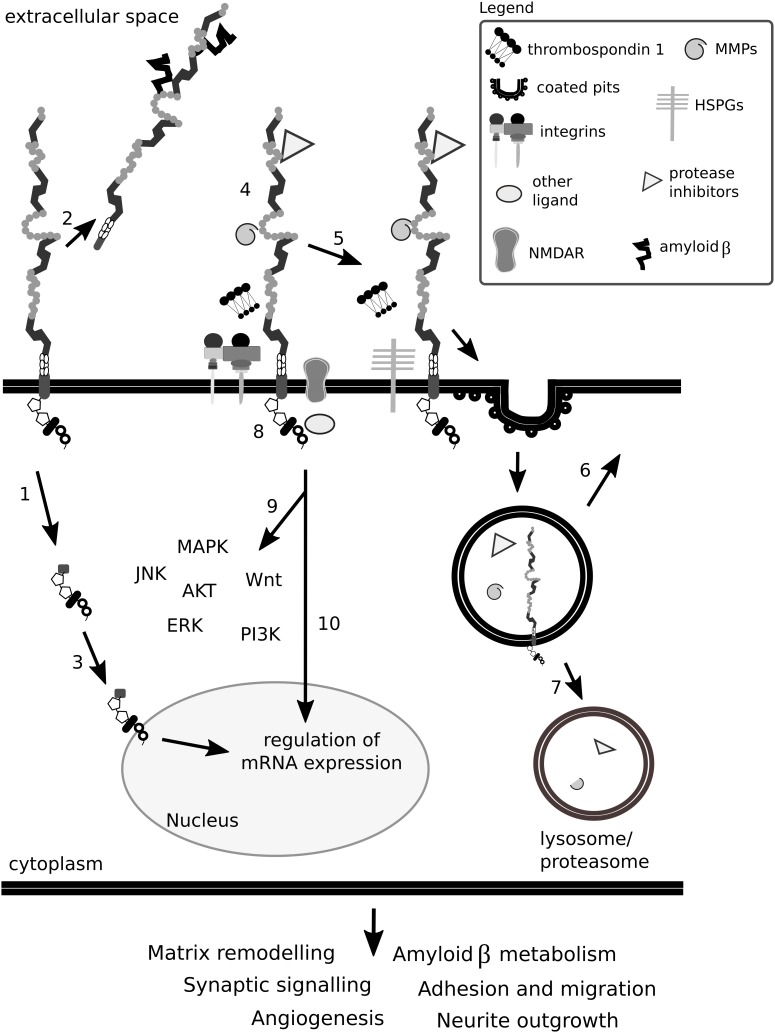
Lrp1 impact on the ECM is complex. Lrp1 can undergo β- and γ-secretase-mediated cleavages that result in two functional receptor domains that exhibit different properties (1). The shed extracellular domain of Lrp1 can interact with ligands located in the matrix and in the circulation (2). The intracellular Lrp1 domain can translocate to the nucleus and regulate the expression of many genes (3). Lrp1 has been reported to interact with more than 40 different molecules, among them matrix metalloproteinases (MMPs) and protease inhibitors, thrombospondins, *N*-methyl-D-aspartate receptors (NMDAR) and integrins (4). Mirroring this multitude, the binding of ligands to Lrp1 can lead to various effects. Ligands can directly bind to the extracellular domain of Lrp1, undergo endocytosis (5) and be transported to lysosomes/proteasomes for degradation (7) while Lrp1 is recycled back to the membrane (6). Some ligands require bridging molecules like thrombospondins or heparan sulfate proteoglycans (HSPGs) to undergo efficient uptake (5). Upon ligand binding to the extracellular and/or cytoplasmic domains of Lrp1, the receptor can become phosphorylated (8), result in the activation of signaling cascades (9) and/or regulate gene transcription (10). Given the above, Lrp1 impacts the ECM either directly or indirectly and influences its remodeling. Lrp1 signaling alters not only the composition of the ECM but also other homeostatic processes like synaptic signaling and angiogenesis, which impact brain functioning.

A general overview of how Lrp1 can interact with the ECM is depicted in Figure 12.

In summary, as the impact of Lrp1 on the ECM is complex and both cell type- and cofactor-dependent, an integrative approach for deciphering Lrp1 function should be implemented for each studied interaction and system.

## Author’s Note

Parts of the review content (especially Figure 1, 10, 11 and the integrin and tPA sections) first appeared in the dissertation thesis (Bres, 2018). This is the only form in which this content has appeared, is in line with the author’s university policy and will be soon available online.

## Author Contributions

EB and AF discussed the concept and content of the review. EB drafted and wrote the manuscript and designed the figures, AF read and revised the manuscript. Both authors agreed to the submitted version.

## Conflict of Interest Statement

The authors declare that the research was conducted in the absence of any commercial or financial relationships that could be construed as a potential conflict of interest. The reviewer DL declared a shared affiliation, with no collaboration, with the authors to the handling Editor at the time of review.
